# Quantitative trait loci analysis and genome-wide comparison for silique related traits in *Brassica napus*

**DOI:** 10.1186/s12870-016-0759-7

**Published:** 2016-03-22

**Authors:** Xiaodong Wang, Li Chen, Aina Wang, Hao Wang, Jianhua Tian, Xiaoping Zhao, Hongbo Chao, Yajun Zhao, Weiguo Zhao, Jun Xiang, Jianping Gan, Maoteng Li

**Affiliations:** Department of Biotechnology, College of Life Science and Technology, Huazhong University of Science and Technology, Wuhan, 430074 China; Provincial Key Laboratory of Agrobiology, Key Laboratory of Cotton and Rapeseed, Ministry of Agriculture, Institute of Industrial Crops, Jiangsu Academy of Agricultural Sciences, Nanjing, 210014 China; Hubei Collaborative Innovation Center for the Characteristic Resources Exploitation of Dabie Mountains, Huanggang Normal University, Huanggang, 438000 China; Hybrid Rapeseed Research Center of Shaanxi Province, Shaanxi Rapeseed Branch of National Centre for Oil Crops Genetic Improvement, Yangling, 712100 China

**Keywords:** *Brassica napus*, Silique traits, QTL, Comparative mapping, Candidate genes

## Abstract

**Background:**

Yield of rapeseed is determined by three components: silique number, seed number per silique and thousand seed weight. Seed number per silique and thousand seed weight are influenced by silique length, seed density, silique breadth, silique thickness and silique volume. Some QTLs for silique traits have been reported in *B. napus*, however, no studies have focused on the six agronomic traits (seed number per silique, silique length, silique breadth, silique thickness, seed density and silique volume) simultaneously, and the genetic determinism of such complex traits have not been fully elucidated.

**Results:**

In this study, the six silique traits were evaluated using 348 lines of a doubled haploid population, the KN population. The results showed that 2, 4, 1, 1 and 2 QTLs explaining > 10 % of phenotypic variation were obtained for silique length, silique breadth, silique thickness, seed number per silique and silique volume, respectively. Notably, three major effect QTLs (*cqSB-C6-1*, *cqSB-C6-2* and *cqSV-C6-3*) were identified in at least three environments, and 17 unique QTLs controlling at least two traits were obtained. A high-density consensus map containing 1225 markers was constructed for QTL comparison by combining the KN map with other five published maps. The comparative results revealed that 14, 13 and 11 QTLs for silique breadth, silique thickness and silique volume might be the potential new QTLs because few QTLs for these traits were reported in *B. napus*. In addition, potential new QTLs for silique length (11), seed number per silique (6) and seed density (5) were also identified. Twenty-five candidate genes underlying 27 QTLs for silique related traits were obtained.

**Conclusions:**

This study constructed QTL analysis in *B. napus*, and obtained 60 consensus QTLs for six silique related traits. The potential new QTLs will enhance our understanding of the genetic control of silique traits, and the stable QTLs provided the targets for improving seed yield in future. These findings provided comprehensive insights into the genetic network affecting silique traits at QTL level in *B. napus*.

**Electronic supplementary material:**

The online version of this article (doi:10.1186/s12870-016-0759-7) contains supplementary material, which is available to authorized users.

## Background

*Brassica napus* (AACC, 2*n* = 38) is cultivated all over the world for the production of vegetable oil, animal feed and biodiesel. At present, developing high-yielding cultivars is one of the most important tasks of rapeseed breeders. Yield of rapeseed is determined by three components: silique number (SN), seed number per silique (SPS) and thousand seed weight (TSW) [1]. SN and SPS determine the total number of seeds per plant. Path analysis identified that TSW has the greatest effect on seed yield, followed by the number of pods per plant [2]. SPS and TSW are influenced by silique length (SL), seed density (SD), silique breadth (SB), silique thickness (ST) and silique volume (SV). Silique related traits showed significant relationships between each other, such as SL has a significant positive relationship with SPS [3, 4]. Therefore, improving seed yield through coordination between these traits is an important breeding goal.

The silique traits are all complex quantitative traits controlled by polygenes and highly influenced by environmental conditions. Application of molecular markers for QTL mapping has proved to be a powerful genetic approach to dissect quantitative traits. In recent years, QTL analysis of silique traits, such as SL, TSW, SPS and SD has been carried out using different populations in *B. napus* [1, 3–6]. More recently, a major QTL on chromosome A9 simultaneously affects TSW and SL in *B. napus* was isolated by fine mapping and association analysis [7]. Li et al. (2015) report the cloning and characterization of *BnaC9.SMG7b* on C9, a major QTL that controls SPS in *B. napus* [8]. In addition, SB, ST and SV are also important silique characteristics for seed yield. Adamskia et al. (2009) found that seed size correlated with oil and protein contents, and large seeds normally have better adaptability during germination [9]. Larger values of SL, SB and ST lead to larger SV, and this may lead to larger seed size and stronger photosynthesis. However, there has been little research concerning SB, ST and SV so far.

Several consensus maps have been constructed by integrating different linkage maps based on common molecular markers [10–13], and QTL hotspots for many agronomic traits in *B. napus* have been identified, including flowering time [14], seed yield [4], oil content [11, 15], heterosis-related traits [16] and plant height [17]. Zhou et al*.* (2014) carried out *in silico* mapping to integrate 1960 QTLs, which increased the density of targeted QTL-linked markers and validated the stable existence of QTLs across different populations [18]. Comparison of QTL position and validation of conserved QTLs through map integration is of great significance for breeders to utilize QTLs effectively.

Although QTLs for silique traits have been identified [10–13], the candidate genes related to these QTLs have not been fully elucidated. Comparative mapping among related species is a powerful tool for genetic studies of transferring genomic information from the well-studied species to those more genetically complicated [19]. Nowadays, much effort focused on comparative analysis between *Brassica* and *Arabidopsis*. For example, Raman et al. (2015) conduct a genome-wide association study for flowering time in *B. napus* and identify seven single nucleotide polymorphism markers were detected within 20 Kb regions of *Arabidopsis* candidate genes, including *BnFLC.A2* accounts for ~23 % of natural variation in diverse accessions [20]. Numerous of predicted genes for other traits were obtained, such as phosphorus homeostasis [21] and SPS [22]. To facilitate the utilization of QTLs for the *Brassica* community, comparative mapping among *B. napus*, *Arabidopsis*, *B. rapa* and *B. oleracea* genomes is necessary to obtain candidate genes, and the released *B. rapa*, *B. oleracea* and *B. napus* genome information may accelerate map-based cloning [23–25].

A large population, a high-density genetic map and replicated experiments in multiple environments are considered as three key factors for increasing statistical power and precision in detecting QTLs [26]. In our previous study, KN doubled haploid (DH) population was derived from a cross between ‘KenC-8’ and ‘N53-2’, which contained 348 lines [11]. This population also showed high phenotypic variation for silique related traits. Using KN DH population, the aim of this study was to detect QTLs for SL, SB, ST, SPS, SV and SD in multiple environments, and to study the relationships among these traits (including TSW). We also constructed a consensus map to perform QTL comparisons between different populations, and identified candidate genes through comparative genome analysis.

## Results

### Trait variation and correlation analysis among different silique traits

**Fig. 1:**
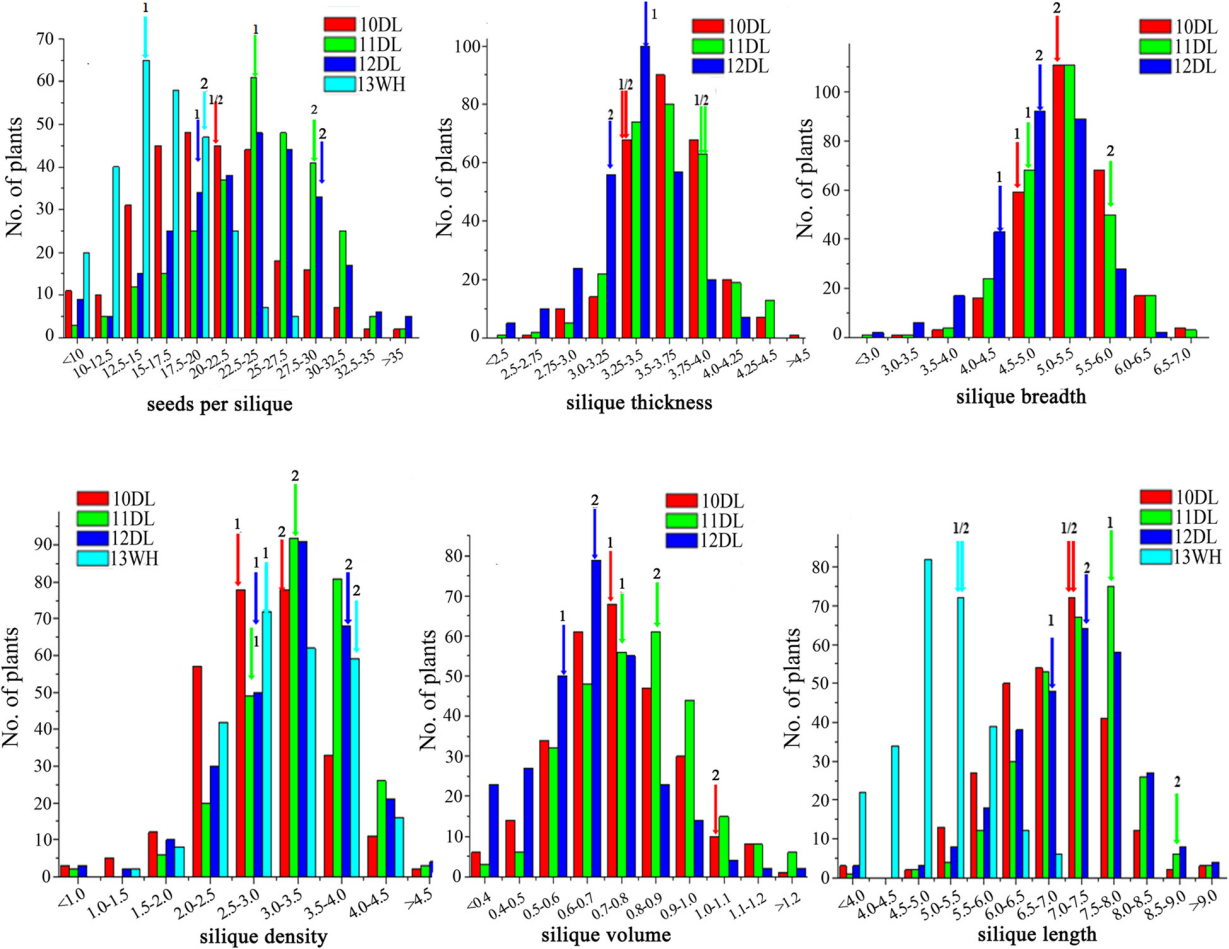
Phenotypic variation of silique traits in KN DH lines and their parents. The numbers 1 and 2 represent KenC-8 and N53-2, respectively. Yield related traits were measured as: silique length (cm), silique breadth (mm), silique thickness (mm), seed per silique, silique volume (ml), seed density (seed number/cm)

The phenotypic performance and frequency distribution of the silique traits in Wuhan (WH, Hubei Province) and Dali (DL, Shannxi Province) were analyzed (Fig. 1). The phenotypic data of these traits all showed continuous distributions. There was high phenotypic variation and transgressive segregation in the KN population, suggested that the segregation pattern of these traits in diverse experiments fitted a normal distribution and were suitable for QTL analysis (Fig. 1, Additional file 1). For instance, KN DH lines had maximum (4.8 mm) and minimum (2.5 mm) values of ST, whereas the ST of parental lines were 3.5 mm and 3.3 mm in 10DL (Additional file 1). However, the parents do not always rank in the same way in some trials, such as the SL in 10DL, 11DL and 12DL environments (Additional file 1), the possible reason was that the phenotypic data of the trait was similar and it had a sampling error.

The results of correlation and linear model analysis among the seven traits are shown in Table 1 and Additional file 2. SL and SPS showed significant positive correlations with five other traits, excluding TSW. SPS and SD had the highest positive correlation coefficient (0.862) among all seven traits. TSW had significant positive correlations with SB, ST and SV, suggesting that TSW could be increased by improving SB, ST and SV. Thus, information on the associations among TSW, SL, SB, ST, SPS, SV and SD is useful for breeders selecting a desirable genotype.

**Table 1:** Phenotypic correlations among silique related traits, seed yield and yield-related traits in KN population

Trait	SL	SB	ST	SPS	SV	SD	TSW	SY	BY	PH	BH	FBN	LMI	PMI
SL	1													
SB	0.26^**^	1												
ST	0.20^**^	0.58^**^	1											
SPS	0.64^**^	0.37^**^	0.12^*^	1										
SV	0.66^**^	0.74^**^	0.61^**^	0.58^**^	1									
SD	0.22^**^	0.27^**^	0.01	0.86^**^	0.29^**^	1								
TSW	0.05	0.40^**^	0.43^**^	-0.18^*^	0.33^**^	-0.26^**^	1							
SY	0.37^**^	0.44^**^	0.30^**^	0.43^**^	0.50^**^	0.36^**^	0.31^**^	1						
BY	0.17^**^	0.51^**^	0.46^**^	0.16^**^	0.47^**^	0.098	0.53^**^	0.83^**^	1					
PH	0.06	0.43^**^	0.42^**^	0.09	0.36^**^	0.07	0.42^**^	0.60^**^	0.74^**^	1				
BH	0.133^*^	0.29^**^	0.31^**^	0.17^**^	0.28^**^	0.14*	0.11	0.37^**^	0.39^**^	0.69^**^	1			
FBN	-0.11	-0.06	0.005	-0.22	-0.11	-0.17	0.06	0.07	0.22^**^	0.12^*^	0.03	1		
LMI	-0.11	0.32^**^	0.29^**^	-0.02	0.195^**^	0.016	0.37^**^	0.41^**^	0.53^**^	0.74^**^	0.32^**^	-0.22^**^	1	
PMI	0.04	0.1	0.12^*^	0.08	0.075	0.135^*^	0.11	0.49^**^	0.37^**^	0.39^**^	0.33^**^	-0.02	0.35^**^	1

*Represent significant at *P* = 0.05; **Represent significant at *P* = 0.01

*SL*; silique length, *SB*; silique breadth, *ST*; silique thickness, *SPS*; seed number per silique, *SV*; silique volume, *SD*; seed density, *TSW*; thousand seed weight, *SY*; seed yield, *BY*; biomass yield, *PH*; plant height, *FBN*; first effective branch number, *LMI*; length of main inflorescence, *PMI*; pod number of main inflorescence

The phenotypic correlations among seed yield and yield-related traits was reported by Zhao et al. (2006) [27]

### QTL mapping for SL, SB, ST, SPS, SV and SD

**Fig. 2:**
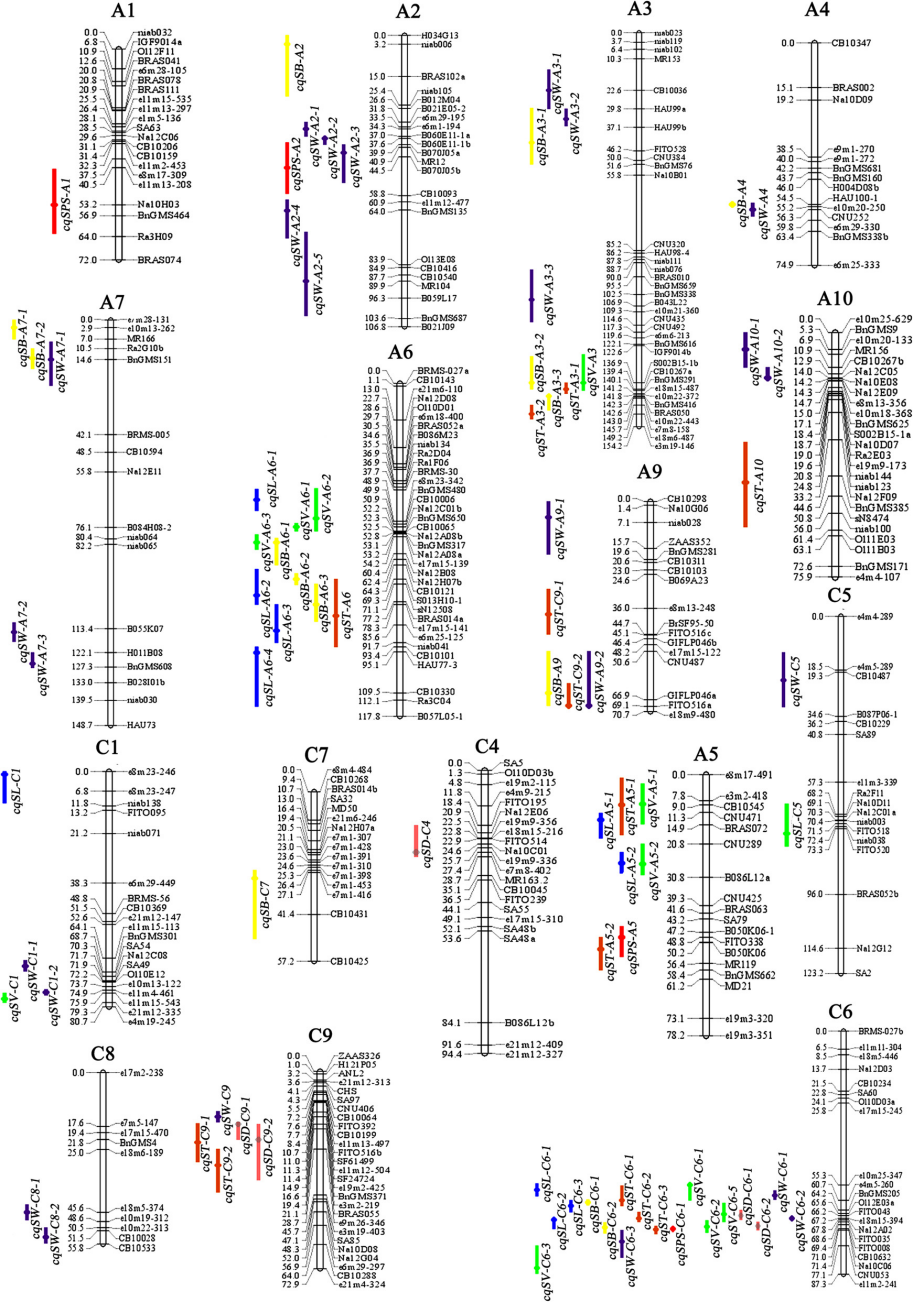
Genetic linkage map and the locations of QTL for silique traits in KN map. The 15 linkage groups with QTLs are represented by vertical bars designated as A1-A10 in A genome and C1-C9 in C genome, based on multiple anchor markers located on each chromosome. The loci names are listed on the right of the linkage groups, and the position of loci is shown on the left side of linkage groups, given in cM. The identified QTLs associated with the seven silique traits are indicated by bars with various backgrounds to the left of each linkage group. (Blue bar, SL; green bar, SV; red bar, SPS; purple bar, TSW; pink bar, SD; yellow, SB; brown bar, ST)

Genome-wide QTL analysis was performed for the SL, SB, ST, SPS, SV and SD and 82 identified QTLs on 15 linkage groups were obtained (Additional file 3). The QTLs detected on each chromosome ranged from one (A1, A4, A10, C4, C5 and C7) to 30 (C6), and 16, 20, 16, 8, 16 and 6 QTLs were obtained for SL, SB, ST, SPS, SV and SD, respectively (Additional file 3 and 4). The identified QTLs with overlapping CIs for the same trait were integrated into a consensus QTL, and then these 82 identified QTLs were integrated into 60 consensus QTLs by meta-analysis (Table 2, Fig. 2). Further analysis showed that 45 were environment-specific QTLs. Six and nine consensus QTLs were identified in three and two microenvironments, respectively (Table 2).

**Table 2:** Consensus QTL obtained for the six silique related traits

QTL^a^	Chr.^b^	LOD^c^	PVE ^d^	Position^e^	CI.^f^	Add.^g^	Environment^h^
*cqSL-A5-1*	A5	8.09	11.04	13.41	11.4-18.9	-0.30	10DL
*cqSL-A5-2*	A5	5.34-6.31	8.57-9.17	26.4	23.25-29.56	-0.27 ~ -0.22	10DL/11DL/13WH
*cqSL-A6-1*	A6	3.56-7.24	5.28-14.36	41.33	37.59-45.06	-0.29 ~ -0.21	10DL/13WH
*cqSL-A6-2*	A6	4.53	5.78	75.11	65.8-78.3	0.25	11DL
*cqSL-A6-3*	A6	4.32	5.63	87.61	78.3-91.7	0.22	11DL
*cqSL-A6-4*	A6	2.94	3.43	95.21	93.4-114.2	0.17	11DL
*cqSL-C1*	C1	2.6	3.68	0.01	0-10.8	0.14	13WH
*cqSL-C5*	C5	3.81	4.68	70.51	69.1-79.5	0.19	11DL
*cqSL-C6-1*	C6	2.86-5.21	4.8-7.39	57.02	54.85-59.19	0.20 ~ 0.39	10DL/11DL/12DL
*cqSL-C6-2*	C6	5.8	7.37	67.81	67.2-71	0.38	11DL
*cqSL-C6-3*	C6	3.76	6.35	62.71	60.7-65	0.26	12DL
*cqSB-A2*	A2	3.01	3.44	3.21	0-22.2	-0.1	10DL
*cqSB-A3-1*	A3	3.24	4.24	43.11	29.6-51.6	0.13	11DL
*cqSB-A3-2*	A3	3.91	4.76	136.91	126.8-139.4	0.13	12DL
*cqSB-A3-3*	A3	3.83	4.65	142.31	141.8-147.7	0.13	12DL
*cqSB-A4*	A4	4.12	5.18	54.51	54.3-54.9	0.13	10DL
*cqSB-A6-1*	A6	4.26	5.62	56.21	54.9-64.3	-0.13	10DL
*cqSB-A6-2*	A6	3.87-5.76	4.73-7.09	69.31	67.31-71.3	-0.15 ~ -0.13	10DL/12DL
*cqSB-A6-3*	A6	5.81	7.16	78.31	71.1-84.3	-0.15	10DL
*cqSB-A7-1*	A7	3.52	4.21	2.91	0-7	0.11	10DL
*cqSB-A7-2*	A7	3.24-5.75	3.99-7.38	14.15	10.51-17.79	0.11 ~ 0.16	10DL/11DL
*cqSB-A9*	A9	5	6.69	64.61	50.6-68.9	-0.15	11DL
*cqSB-C6-1*	C6	7.43-9.61	9.42-15.06	61.43	60.47-62.39	0.20 ~ 0.27	10DL/11DL/12DL
*cqSB-C6-2*	C6	7.96-11.25	9.92-14.84	70.59	68.69-72.5	0.20 ~ 0.26	10DL/11DL/12DL
*cqSB-C7*	C7	3.22	4.1	29.11	26.4-49.6	0.11	10DL
*cqST-A3-1*	A3	7.35	9.13	139.41	136.9-141.3	0.1	11DL
*cqST-A3-2*	A3	3.25	4.89	149.31	145.7-151.3	0.08	12DL
*cqST-A5-1*	A5	6.08	7.52	9.11	1-18.1	-0.1	11DL
*cqST-A5-2*	A5	4.49	6.21	52.21	48.8-58.4	0.09	11DL
*cqST-A6*	A6	3.08	3.9	82.31	69.3-93.4	-0.07	11DL
*cqST-A9-1*	A9	7.37	10.41	38.01	29.6-44.7	-0.1	10DL
*cqST-A9-2*	A9	5.75	7.15	68.91	61.3-69.2	-0.1	11DL
*cqST-A10*	A10	3.14	4.18	46.61	34.1-60.4	-0.07	10DL
*cqST-C6-1*	C6	4.84	5.83	60.71	55.6-62.7	0.09	11DL
*cqST-C6-2*	C6	3.93	4.74	67.21	65-68.6	0.08	11DL
*cqST-C6-3*	C6	3.01-4.31	4.14-5.81	71.31	69.95-72.66	0.08 ~ 0.09	11DL/12DL
*cqST-C9-1*	C9	2.83-4.43	3.26-6.16	26.29	19.22-33.37	-0.09 ~ -0.08	10DL/11DL
*cqST-C9-2*	C9	4.56	7.58	34.71	28.7-44.7	-0.06	10DL
*cqSPS-A1*	A1	3.16	4.69	53.21	41-62.9	0.94	13WH
*cqSPS-A2*	A2	3.69	5.52	48.51	39.3-58	-1.51	12DL
*cqSPS-A5*	A5	3.46	5.47	48.81	45.4-54.6	-0.99	13WH
*cqSPS-C6-1*	C6	6.08	9.82	57.31	55.2-59.3	2.12	12DL
*cqSPS-C6-2*	C6	6.74	9.26	69.41	69.3-71.0	2.16	12DL
*cqSPS-C6-3*	C6	2.87-7.99	4.32-11.16	71.01	70.22-71.8	1.56 ~ 2.02	11DL/12DL/13WH
*cqSV-A3*	A3	3.59	4.79	136.91	125.9-140.1	0.04	12DL
*cqSV-A5-1*	A5	5.62-6.43	6.51-8.48	8.78	2.76-14.81	-0.05	10DL/11DL
*cqSV-A5-2*	A5	3.99	5.26	26.81	20.8-29.9	-0.04	10DL
*cqSV-A6-1*	A6	2.73	3.47	47.71	37.4-52.2	-0.04	11DL
*cqSV-A6-2*	A6	2.93-3.34	3.79-3.86	50.9	49.47-52.33	-0.04	10DL/12DL
*cqSV-A6-3*	A6	2.60-2.65	3.33-3.43	56.2	53.47-58.92	-0.03	10DL/11DL
*cqSV-C1*	C1	3.38	4.36	77.91	75.9-79.3	-0.04	11DL
*cqSV-C6-1*	C6	6.2	7.59	55.31	54.8-60.7	0.05	10DL
*cqSV-C6-2*	C6	5.71	8.15	65.71	61.7-68.6	0.06	12DL
*cqSV-C6-3*	C6	6.09-8.47	8.26-10.39	70.16	67.97-72.34	0.06-0.07	10DL/11DL/12DL
*cqSV-C6-4*	C6	6.2	8.25	85.11	77.1-87.1	0.05	10DL
*cqSD-C4*	C4	3.03	4.36	27.41	18.4-28.7	0.15	10DL
*cqSD-C6-1*	C6	3.12	4.47	66.21	64.2-67.8	0.15	11DL
*cqSD-C6-2*	C6	2.98-4.39	4.28-6.15	70.02	68.52-71.52	0.17	11DL/12DL
*cqSD-C9-1*	C9	2.87-4.62	5.17-6.71	19.41	18.6-25.3	0.27	10DL
*cqSD-C9-2*	C9	2.87	5.17	25.11	19.4-40.2	0.16	13WH

^a^QTL name, two letters followed the ‘*cq*’ indicate different traits (*SL*; silique length, *SB*; silique breadth, *ST*; silique thickness, *SPS*; seed number per silique, *SV*; silique volume, *SD*; seed density); ^b^Location of QTLs; ^c^ LOD score of consensus QTLs; ^d^Phenotype variation explanation of QTLs; ^e^Peak position; ^f^Confidence interval; ^g^ Additive effects; ^h^Environments of consensus QTLs appeared

Sixteen identified QTLs for SL were detected with the phenotypic variation explained (PVE) in the range of 3.4 %–14.36 % (Additional file 3). The 16 identified QTLs were integrated into 11 consensus QTLs (Table 2, Fig. 2), and two (*cqSL-A5-2* and *cqSL-C6-1*) and one (*cqSL-A6-1*) consensus QTLs were detected in three and two microenvironments, respectively. Among these identified QTLs, QTL *qSL-A6-2* showed the largest PVE of 14.36 % and had the LOD value of 7.24 (Additional file 3). In order to identify its effect, genotypes of the DH population were collected for the closely linked marker CB10006 on A6 of this QTL. In 10DL and 13WH, DH lines carrying the ‘N53-2’ (A) had an average length of 6.75 ± 0.77 and 4.95 ± 0.44, while DH lines carrying the ‘KenC-8’ (B) had an average length of 6.94 ± 0.83 and 5.05 ± 0.60, respectively. There was no significant difference when comparing lines carrying ‘B’ with lines carrying ‘A’ (Table 3), and the non difference between the alleles based on CB10006 could be due to recombination between the markers and the causative genes.

**Table 3:** Effect analysis of QTLs relative higher phenotype variance explanation for the silique related traits

Environment	CB10006	Additive	N^a^	SL^c^		
	(A6)			Range	Mean	P^b^
10DL	A	-0.21	161	2.9-9.16	6.75 ± 0.77	0.1
	B		97	3.1-9.7	6.94 ± 0.83	
13WH	A	-0.29	160	4.12-5.42	4.95 ± 0.44	0.27
	B		96	2.44-6.8	5.05 ± 0.60	
Environment	e4m5-260	Additive	N^a^	SB		
	(C6)			Range	Mean	P^b^
10DL	A	0.21	184	2.64-4.50	3.67 ± 0.07	0.19
	B		82	2.68-4.36	3.60 ± 0.15	
11DL	A	0.20	184	2.54-4.22	3.58 ± 0.10	0.26
	B		82	2.42-4.02	3.52 ± 0.10	
12DL	A	0.27	184	2.34-4.22	3.36 ± 0.09	0.37
	B		82	2.42-4.02	3.31 ± 0.16	
Environment	e8m13-248	Additive	N^a^	ST		
	(A9)			Range	Mean	P^b^
10DL	A	-0.10	140	2.5-4.22	3.57 ± 0.07	0
	B		128	2.92-4.8	3.73 ± 0.11	
Environment	CB10632	Additive	N^a^	SPS		
	(C6)			Range	Mean	P^b^
11DL	A	2.02	187	11-35	25.04 ± 22.05	0
	B		79	1-39	21.66 ± 37.39	
12DL	A	1.56	187	4-38	23.94 ± 31.95	0
	B		79	1-40	19.68 ± 47.00	
13WH	A	1.56	187	4-37	20.51 ± 34.25	0.03
	B		79	2-31	18.89 ± 28.45	
Environment	Na12A02	Additive	N^a^	SV		
	(C6)			Range	Mean	P^b^
10DL	A	0.06	168	0.20-1.14	0.79 ± 0.03	0
	B		59	0.32-1.12	0.68 ± 0.02	
11DL	A	0.07	168	0.20-1.70	0.83 ± 0.04	0
	B		59	0.34-1.14	0.71 ± 0.02	
12DL	A	0.06	168	0.14-1.24	0.69 ± 0.03	0
	B		59	0.14-1.62	0.59 ± 0.05	

^a^Number of DH lines for each group (N53-2: A; KenC-8: B) classified according to the genotype of the markers that closely linked to the major QTL; ^b^ *P* values obtained by *t* test among groups (0.05 level)

^c^See Table 1 for abbreviations

For SB, 20 identified QTLs were obtained with PVE ranging from 3.44 % to 15.06 % (Additional file 3). Finally, these 20 identified QTLs were integrated into 14 consensus QTLs, including each two consensus QTLs were detected in three and two microenvironments (Table 2, Fig. 2). QTLs *cqSB-C6-1* and *cqSB-C6-2* with PVE >10 % were considered as major QTLs, as they were repeatedly detected in three microenvironments (10DL, 11DL and 12DL). Among the identified QTLs, *qSB-C6-3* had the highest PVE and had a relatively high LOD value of 9.61. To identify its effect, genotype data of the DH population for the nearest marker e4m5-260 on C6 was collected, and a similar result for SL was observed (Table 3).

For ST, 16 identified QTLs were obtained with PVE of 3.26 %–10.41 % (Additional file 3). The 16 QTLs were integrated into 13 consensus QTLs, and two QTLs were detected in two microenvironments (Table 2, Fig. 2). QTL *qST-A9-1*, with the highest PVE of 10.41 %, was closely linked with marker e8m13–248 on A9, and the phenotypic data of DH lines carrying ‘A’ alleles was significantly smaller than lines carrying ‘B’ alleles, suggesting that alleles responsible for increasing ST existed in the male parent ‘KenC-8’ (Table 3).

For SV, 16 identified QTLs with PVE of 3.33 %–10.39 % were obtained (Additional file 3). The 16 identified QTLs were finally integrated into 11 consensus QTLs, including one and three QTLs were detected in three and two microenvironments, respectively (Table 2, Fig. 2). QTL *cqSV-C6-3* that integrated from *qSV-C6-3* (PVE of 10.14 %), *qSV-C6-4* (10.39 %) and *qSV-C6-5* (8.26 %) was considered as a major QTL. Further investigation revealed that the phenotypic data of lines carrying ‘A’ and ‘B’ alleles of tightly linked marker Na12A02 on C6 (*qSV-C6-4* with the highest PVE) showed significant differences. The average SV was significantly larger in lines carrying ‘A’ alleles than ‘B’ alleles, suggesting that the genes responsible for increasing SV exist in the female parent ‘N53-2’ (Table 3).

Eight QTLs for SPS with PVE within the range of 4.32 %–11.16 % were obtained (Additional file 3), and then were integrated into six consensus QTLs. QTL *cqSPS-C6-3* was detected in three environments (Table 2, Fig. 2), which was integrated from *qSPS-C6-3*, *qSPS-C6-4* and *qSPS-C6-5* with PVE of 4.32 %, 11.16 % and 5.94 %, respectively. QTL *qSPS-C6-4* (with the highest PVE) was closely linked with marker CB10632 on C6, and further analysis revealed that the related phenotypic data of lines carrying ‘A’ alleles had significantly more SPS, on average, than lines carrying ‘B’ alleles. This suggests that the genes increasing SPS existed in the male parent ‘N53-2’ (Table 3).

For SD, six QTLs were identified with PVE of 4.28 %–6.71 % (Additional file 3), none of these were considered as major QTL. The six identified QTLs were integrated into five consensus QTLs. Only *cqSD-C6-2* was detected in two microenvironments (Table 2).

Forty-two QTLs for TSW were detected in eight experiments, and 26 consensus QTLs were obtained, and three and six consensus QTLs were detected in four and two microenvironments, respectively (Additional file 3) [27]. The PVE of these QTLs were within the range of 3.15 %–19.62 %. QTLs *cqSW-A7-2* and *cqSW-C1-1* were two major QTLs with PVE >10 % in two different experiments, respectively.

### Detection of pleiotropic unique QTLs for silique related traits

**Fig. 3:**
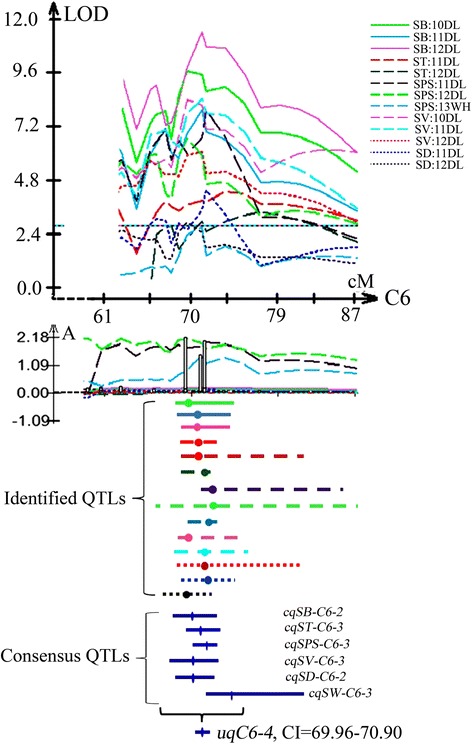
An example of the unique QTL classification on C6 chromosome. Identified QTLs for different silique traits in different experiments are shown by curves above the line of linkage group, and their additive effect are shown by curves of the same color below the line of the linkage group. The CIs of identified QTLs are shown by the same type of lines with curves. The solid blue lines are the CIs of the integrated QTLs by meta-analysis. A additive effect

There were significant phenotypic correlations among the seven traits (SL, SB, ST, SPS, SV SD and TSW), which was also reflected by the genomic location and the effects of the corresponding QTLs. A trait-by-trait meta-analysis revealed that the 86 consensus QTLs for the seven traits (including TSW) were integrated into 52 unique QTLs (Additional file 5). Among them, 17 unique QTLs have pleiotropic effects and control at least two traits (Table 4). These unique QTLs were generally classified into two kinds: unique QTLs integrated from QTLs with the same direction of additive effect, and QTLs with opposite additive effect directions (Additional file 5). For instance, *uqC6-4* obtained from QTLs controlling six silique related traits except for SL with positive additive effect (Fig. 3), which was in accordance with the significant positive correlations among these traits. However, *uqA2-4* belonged to the second kind, the additive effect of *cqSPS-A2* (–1.51) was negative while *cqSW-A2-3* (0.12) was positive (Additional file 5), which might explain the negative phenotype correlation between SPS and TSW. In addition, QTL *uqC6-1* was integrated from QTLs controlling SV, SL, TSW and SPS that exhibited the same direction of parental contribution. This result might explain the negative but low correlation between SPS and TSW, and the positive but low correlation between SL and TSW.

**Table 4:** The list of 17 pleiotropic unique QTL for silique related traits in KN population

Name	Chr.	Trait^a^	Position^b^	CI.^c^
*uqA2-4*	A2	SPS/SW	44.98	39.38-50.58
*uqA3-2*	A3	SB/SW	34.57	31.41-37.73
*uqA3-4*	A3	SB/SV/ST	138.96	136.96-141.62
*uqA5-1*	A5	SV/ST/SL	11.75	8.76-14.73
*uqA5-2*	A5	SV/SL	26.53	23.94-29.12
*uqA5-3*	A5	ST/SPS	50.43	47.11-53.75
*uqA6-3*	A6	SV/SB	56.20	53.84-58.55
*uqA6-5*	A6	SL/SB	76.62	72.08-81.16
*uqA6-6*	A6	SL/ST	86.35	80.50-92.21
*uqA7-2*	A7	SB/SW	14.22	10.91-17.53
*uqA9-3*	A9	SB/ST/SW	68.32	64.94-71.70
*uqC6-1*	C6	SV/SL/SW/SPS	57.57	56.52-58.63
*uqC6-2*	C6	ST/SB/SL	61.58	60.73-62.44
*uqC6-3*	C6	SV/SD/SW/ST/SL	67.10	66.50-67.70
*uqC6-4*	C6	SPS/SD/SV/SB/ST/SW	70.43	69.96-70.90
*uqC9-1*	C9	SW/SD	17.33	15.62-19.04
*uqC9-2*	C9	SD/SW/ST	26.06	21.57-30.55

^a^See Table 1 for abbreviations; ^b^Peak position; ^c^Confidence interval

### Consensus map construction and QTL comparison

Five mapping populations (SS, BE, QN, TN and HY) were selected for construction of consensus map and QTL comparison (Additional file 6). For exact comparisons among populations, QTLs collected in each population were used in the first round of meta-analysis to obtain consensus QTLs (Additional file 6). The KN DH map was treated as a reference map, and the markers and QTLs were projected from the five chosen populations onto the KN map. Finally, a consensus map containing 1225 markers was constructed, with the exceptions of A9, C4, C7 and C8 that lacked a common marker (Additional file 6 and 7). A total of 117 consensus QTLs, including 76 on the A genome and 41 on the C genome, were located from the chosen five populations (Additional file 6). Finally, 34 QTLs (26 in the A genome and eight in C genome), including 7, 1, 1 and 25 QTLs controlling SL, SD, SPS and TSW were aligned to the consensus map, respectively (Additional file 8). The number of aligned QTLs on each chromosome ranged from one (A6, A10 and C9) to 11 (A3).

**Fig. 4:**
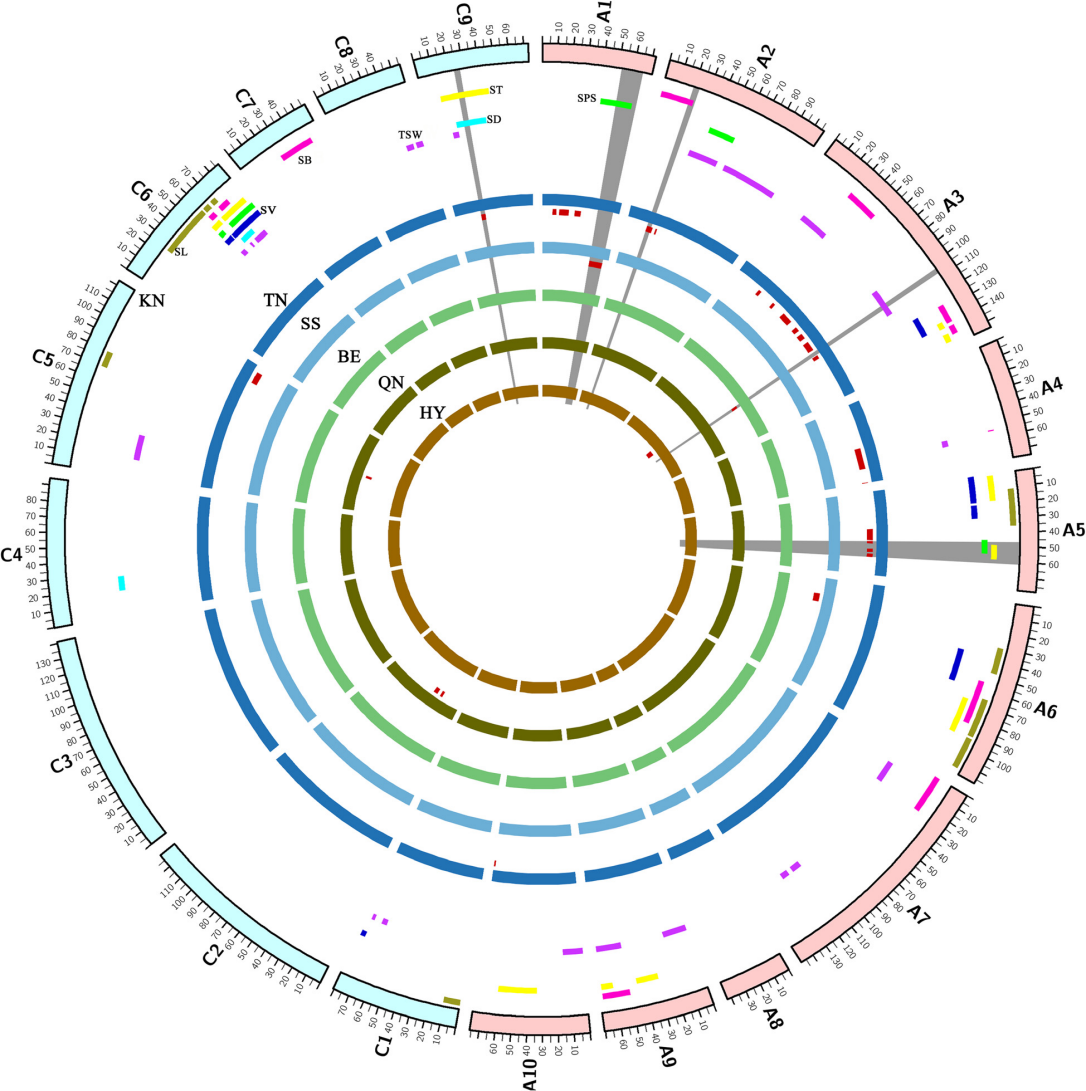
QTL comparison of the seven traits among different populations. QTLs obtained in KN DH population are shown on the outside circle. The inside circles represent QTLs projected from the TN, SS, BE, QN and HY populations, respectively. Different traits are indicated by bars with various backgrounds (*Green bars*, QTLs for seed number per silique; *blue bars*, silique volume; *purple bars*, thousand seed weight; *red bars*, silique breadth; *yellow bars*, silique thickness; *yellowish green bars*, silique length). The grey traits indicated that QTLs detected in different population were co-localized

For TSW, QTLs with overlapping CIs among different populations were observed on A2, A3 and A4 (Additional file 7). For the other six traits, no common QTLs were detected among the different populations. On chromosome A1, four QTLs were projected from TN and SS populations. QTLs *SS-qSL-A1* and *cqSPS-A1* were co-localized in the CI of 52.1–62.9 cM. On chromosome A2, three QTLs were projected from TN onto KN, and QTL *TN-qTSW-A2-1* had an overlapping CI with *cqSB-A2*. Eleven QTLs were projected from TN, HY and BE onto KN on A3. Among them, *TN-qTSW-A3-1* and *cqSW-A3-1*, and *TN-qTSW-A3-2* and *cqSB-A3-1* were co-localized with overlapping CIs. QTLs *TN-qTSW-A3-8*, *TN-qTSW-A3-9* and *BE-cqSPS-A3* had overlapping CIs with *cqSW-A3-3*. On chromosome A4, two QTLs were projected from the TN population and *TN-qTSW-A4-1* co-localized with *cqSB-A4* and *cqSW-A4*. Four QTLs on chromosome A5 were projected from TN onto the consensus map, with *cqSPS-A5* and *TN-qTSW-A5-2*, and *cqST-A5-2* and *TN-qTSW-A5-3* co-localized. On chromosome C9, one QTL was aligned from TN with an overlapping CI with *cqSD-C9-2* and *cqST-C9-1*. Two QTLs were projected from the QN population on chromosome C2, while no QTL was detected on C2 in the KN population (Fig. 4). In addition, on A6, A10, C1 and C5, SS, TN, QN and QN had 1, 1, 2 and 3 projected QTLs, respectively, and all of them differed from the QTLs detected in the present study. Compared with the abovementioned five populations, 14, 13 and 11 consensus QTLs might be the first detected for SB, ST and SV, while 11, 6, 5 and 20 of the 47 consensus QTLs for SL, SPS, SD and TSW were potential new QTLs, respectively (Fig. 4). Especially for SL and SD, QTLs on A5 and C4 might be the first detected. These conserved QTLs repeatedly detected in different populations might provide target QTLs for breeders and map-based cloning of genes contributing to these silique traits in *B. napus*, and newly detected QTLs might increase our knowledge of the genetic mechanisms of these traits.

### Comparative genome analysis and candidate gene identification

On the basis of the distribution of 24 conserved chromosomal blocks of *Arabidopsis* described by Schranz et al. [28], 33 blocks and 127 islands in the KN DH map were identified with respect to the *Arabidopsis* genome according to 141 markers with known sequence information [27].

All of the QTLs with CIs containing homologous genes were separately compared to the physical genomic regions of *B. rapa* (A genome) and *B. oleracea* (C genome) (Additional file 9). Based on the map alignment between *Arabidopsis* and *B. napus*, a total of 88 orthologs of 37 genes that controlled these silique traits in *Arabidopsis* were mapped onto the synteny blocks and islands (Additional file 10), and 25 genes were located in the CI of 27 QTLs (Additional file 9 and 11). Among the 25 genes, several were shown to have a relationship with the seven silique traits, including *EXS*, *GASA4*, *ANT*, *TTG2* and *AP2*. Especially on chromosome C6, gene *ATAGP19* (*BnaA07g24460D*) and *CUL3B* (*scaffold_213*) were associated with unique QTLs controlling SV, SD, ST and SW. The *ATAGP19* mutant could lead to fewer siliques, less seed production and several abnormalities in cell size, number, shape and packing [29]. *CUL3B* is essential for normal embryogenesis [30]. At the same time, sequences of these genes available for *B. rapa* and *B. oleracea* were also mapped onto related chromosomes (Additional file 10).

## Discussion

In the present study, the genetic basis for the six silique traits (SPS, SL, SB, ST, SD and SV) at QTL level was performed in the KN DH population containing 348 lines, possibly the largest population yet used to perform QTL mapping for silique traits. SL is known to be positively correlated with SPS [31, 32], and the same result was also obtained in the present study. No significant positive relationship was observed between SL and TSW, which differed from previous results [1, 2]. The most persuasive explanation for the phenomenon was that the population used in the present study was different from other published populations, and each mapping population might carry different alleles for silique related traits and represent its own genetic background. For example, Yang et al. (2012) indicated that SL was significantly correlated with SW, using a RIL derived from a cross between an EMS mutant with extremely long SL and SW, and an inbred line with regular SL and SW [1]. By comparison, the KN population for SL and SW showed high phenotypic variation but the two parents showed no significant difference. To date, little research has been conducted on SB, ST and SV and their relationships with other yield-related factors. In this study, SL, SB and SV all showed significant positive relationships with SPS and SD (Table 1). The present results revealed that SPS was significant negatively correlated with TSW, as also observed by Zhang et al. (2011) [3]. It is noteworthy that all the six silique traits in the present study showed highly and positively correlated with the level of seed yield (Table 1), suggesting that at least some of the underlying genetic determinism or same genes are shared across the formation and development of these traits. These silique traits colud be served as important traits for section of high yield cultivar for breeding purpose. These results suggest that coordinating the balance among these agronomic traits of *B. napus* was the premise of ensuring the high and stable yield production.

Fine mapping and map-based cloning are efficient ways to dissect the genetic bases of quantitative traits and to identify genes underlying these traits. QTLs with large PVE and can be detected in different environments are more suitable for fine mapping and map-based cloning [18]. Integrating QTL results across different environments can determine which QTLs are less influenced by environmental factors, and can help to isolate environment-specific QTLs [18]. In the present study, 15 stable consensus QTLs expressed in at least two microenvironments, and 45 environment-specific QTLs were obtained (Table 2). The high proportion of environment-specific QTLs suggested a large impact of the natural environment on silique-associated traits. Most of them had minor effects with low LOD scores. It is clear that use such minor QTLs for MAS or clone such QTLs *via* fine mapping is unlikely. However, these QTLs provide an important genetic resource for further research [17]. The explanation for the existence of stable QTLs was that they could be responsible for main genetic effects with high LOD scores, as QTLs with major effects are more likely to be stable across multiple environments [33-35]. In the present study, three QTLs with PVE > 10 % controlling SB (*cqSB-C6-1* and *cqSB-C6-2*) and SV (*cqSV-C6-2*) were identified in at least three microenvironments in DL (Table 2). In addition, *cqSB-C6-1* and *cqSB-C6-2* were both integrated from three identified QTLs detected in DL and with mean additive effects of 0.22 and 0.22, respectively (Additional file 3). These results showed that when alleles from ‘KenC-8’ existed in the two QTLs, the SB could increase about 0.8 mm in DL*.* These kinds of QTLs might be worthy of attention when doing MAS for developing varieties with special adaptability. In addition, unique QTL *uqC6-4* would be the most interesting for breeding. The *uqC6-4* could control as much as six traits except for SL, which were all with positive additive effect. Compared with the seed yield and oil content QTLs detected in the same population, *uqC6-4* was co-localized with the major QTL for seed yield (*cqSY-C6-2*), which was repeatedly detected in three environments [27], and was also co-localized with the QTL (*qOC-C6-4*) for seed oil content consistently expressed in four environments [11]. Among the two parents of KN DH population, ‘KenC-8’ was the parental of the variety ‘Zayou59’ released in 1996 in China, and ‘N53-2’ was a DH line with seed oil content >50 % [11]. QTLs detected in KN population could be used in breeding, not only for developing high-yielding cultivars, but also for increasing seed oil content.

Although the genetic determinism of these silique traits remain largely unknown in rapeseed, correlation analysis with the same DH population may provide some insights. Most pairs of traits showed significant phenotypic correlations in this study, which was also reflected by the genome location and effects of QTL detected. Many QTL clusters were observed on chromosomes A2, A3, A5, A6, A7, A9, C6 and C9 (Table 4). With the overlapping CIs of QTLs controlling different traits, 17 unique QTLs that controlled at least two traits were obtained and the unique QTL (*uqC6-4*) could control as much as six traits (Fig. 3). Co-localizations between QTLs for some traits were expected such for SB/ST/TSW due to the correlation (Table 1). This were also in agreement with the pleiotropic unique QTLs, such as *uqA9-3* and *uqC6-4* control the three traits simultaneously, and *uqA3-2* and *uqA7-2* have effect on SB and TSW, and *uqA3-4* and *uqC6-2* effect SB and ST simultaneously, and *uqC9-2* have effect on ST and SW (Additional file 5). Previous studies on silique traits also demonstrated the existence of pleiotropic QTLs in *B. napus* [1, 4]. In addition, the consistency of QTL loci on the chromosome for various traits may facilitate selection efficiency by selecting markers closely associated with these traits. Furthermore, if the direction of the additive effect of the QTLs were the same, selection would be easier and more effective, such as for QTLs clustered in the same region of C6 (Additional file 5).

In general, a consensus map is a powerful tool to validate conserved QTLs across populations. To compare QTLs detected in different populations, a consensus map containing 1225 markers was constructed (Additional file 6). To our knowledge, there are few reports concerning QTL analysis for SB, ST and SV, and the 14, 13 and 11 consensus QTLs in the present study might be new QTLs. Compared with 34 projected QTLs from other five maps, three QTLs for TSW were the same as those identified in previous studies, including *cqSW-A3-1*, *cqSW-A3-3* and *cqSW-A4*. In addition, 11, 6 and 5 consensus QTLs for SL, SPS and SD observed in the present study were differed from those of the other five populations, and hence might considered as potential new QTLs. The detected QTLs for SL had very high PVE, reaching 53.4 % and 54.4 % on A9 [1, 36], while no QTL for SL was obtained on A9 in the present study, possibly due to only minor differences in SL between the two parents. Comparison among different populations would also facilitate an assessment of intraspecific variation for these QTLs.

The establishment of comparative mapping between *Arabidopsis* and *B. napus* is a powerful tool to identify candidate genes underlying QTLs [5]. In this study, 25 candidate genes were assigned to the CIs of 27 QTLs (Additional file 11), and several of these genes were shown to have a relationship with the six silique traits. Studies have revealed that the genes *ANT*, *ARF2* and *TOC1* underlying QTLs exist on chromosomes A1–A3 [1, 18, 36], whereas *ANT* underlying QTLs aligned on A3 and C1 was observed in the present study. Fan et al. (2010) detected QTLs for TSW on A7, which might correspond to *TTG2* and *MINI3* [37], whereas *TTG2* underlying QTLs was aligned on A4 and A5 in the present results. As well as *ANT*, *TTG2* and *MINI3*, gene *EXS* that controlled embryo development was aligned in the CIs of QTLs controlling SPS [38]. *GASA4*, which positively affected both seed size and total seed yield, was located in the CI of a QTL controlling TSW [39]. *AP2* was found underlying QTLs controlling TSW on A2 and SL on C1, and *ap2* mutants could produce larger seeds than wild type [40]. On A5, *IKU1* controlling seed size was located in the CI of QTLs controlling SPS and ST [41]. Genes *FIS2* and *MEA* were aligned in the CIs of QTLs controlling SPS and ST on A5 and QTLs controlling SL and ST on A6, respectively. These two genes repressed seed development in the absence of pollination [42]. On C5, *CUL3A* was associated with QTLs controlling SL, which is essential for normal embryogenesis [30]. *ATAGP19* and *CUL3B* were associated with unique QTLs controlling SV, SD, ST and SW; and the *AtAGP19* mutant had fewer siliques, less seed production and several abnormalities in cell size, number, shape and packing [29]. Compared with currently published *B. napus* genome information, related *B. napus* genome information was also obtained. These findings provided useful resources on candidate genes for seed yield improvement.

## Conclusions

We identified 82 individual QTLs for six silique traits (SPS, SL, SB, ST, SD and SV), and then integrated these QTLs into 60 consensus QTLs by meta-analysis. Among them, all of the 14, 13 and 11 consensus QTLs for SB, ST and SV might be the potential new QTLs because few QTLs for these traits were reported in *B. napus*. After compared with five published populations, 11, 6 and 5 potential new consensus QTLs for SL, SPS and SD were obtained. By *in silico* mapping analysis, we identified 25 candidate genes underlying 27 QTLs for silique related traits. Our results provided useful information into the regulatory model for the control of silique in *B. napus*.

## Methods

### Plant material and genetic linkage map

In this study, a segregating DH population, named KN DH, from a cross between male ‘KenC-8’ and female ‘N53-2’ was used to detect QTLs for silique traits. The KN DH population consisted of 348 lines, which was previously used for developing the linkage map (KN map) and QTL evaluation for oil content [11]. The KN genetic linkage map was constructed with 403 molecular markers, including 275 simple sequence repeats, 117 sequence-related amplified polymorphisms, 10 sequence tagged sites and one intron fragment length polymorphism, which covered a total length of 1783.9 cM. The genotype of the population had two kinds of bands, the genotypes of the two parents: ‘KenC-8’ (B) and ‘N53-2’ (A). If the additive effect of QTLs was negative, this suggested the increasing allele originated from ‘KenC-8’, and otherwise it was from ‘N53-2’.

### Field experiment and data collection

The field experiments were carried out in two locations: Wuhan in the Hubei Province (a semi winter-type rapeseed growing area, coded WH) and Dali in the Shannxi Province (a winter-type rapeseed growing area, coded DL). The seeds were sown in 2009–2011 in DL and 2012 in WH; and the related experiments were named 10DL, 11DL, 12DL and 13WH, respectively. The experiment location of WH was the experiment base of Huazhong University of Science and Technology, and DL was the experiment base of Hybrid Rapeseed Research Center of Shaanxi Province. No specific permissions were required for the field trials. Year–location combinations were treated as microenvironments, and then these microenvironments were divided into two contrasting macroenvironments: semi-winter and winter. For SL, SPS and SD, phenotypic data were collected in all of the four microexperiments. For SB, ST and SV, phenotypic data were collected in three microenvironments: 10DL, 11DL and 12DL. For TSW, phenotypic data was used to detect its relationships with the above six silique traits, and the QTLs for TSW obtained by Zhao et al. (2016) in eight microenvironments were used for detecting of unique QTLs and QTL comparison in the present study (Additional fine 3) [27]. For each trial, all 348 lines together with their parents were grown in a completely randomized block design with two replications in WH, and three replications in DL. Each line was grown in two rows per plot in all three locations, every row contained about 12 plants, and the distance between rows and between plants was 0.4 and 0.2 m, respectively. The field management followed normal agricultural practice.

All DH lines were open-pollinated in all environments to keep a full record of fertilization rates. At the mature stage, five representational plants in the middle of each plot were selected to analyze the six silique traits. Then, six well-developed siliques, randomly selected from the first branch adjacent to the main inflorescence, were used to investigate phenotypic data. As the rapeseed silique is oval-shaped, the maximum width of a silique in the middle position was treated as “breadth”, and the minimum-data at the same position was treated as “thickness”. Phenotypic data were collected as follows. (1) SL (cm): estimated from the average length (excluding the length of beak) of the six well-developed chosen siliques. (2) SB and ST (both mm): estimated from the average breadth and thickness, respectively, of the six chosen siliques using a Vernier caliper. (3) SPS: estimated from the average value of six chosen siliques. (4) SV (mL): estimated from the average volume of the six chosen siliques using a cylinder containing water, from the volume change following submersion of siliques [43]. (5) SD: measured according to SD = SPS/SL. All phenotype data were collected in three measurements per replication.

### QTL analysis and meta-analysis for the silique traits

Basic statistical analysis was implemented using SPSS 18.0 software (SPSS Inc., Chicago, IL, USA). QTL analysis for SL, SB, ST, SPS, SV and SD were performed by the QTL Cartographer 2.5 software with composite interval mapping (CIM) [44]. A walking speed was set to 2 cM and a window size of 10 cM with five background cofactors. The LOD threshold was determined by 1000-permutation test based upon a 5 % error rate [45], and a LOD of 2.7–3.2 was used to identify the existence of QTLs in each environment, and these QTLs were termed ‘identified QTLs’. The method for QTL nomenclature was as described by Wang et al*.* (2013) [11]. A number was added if more than one QTL was located on the same chromosome, for instance, *qSL-A5-2*.

Identified QTLs repeatedly detected in different microenvironments and with overlapping CIs for the same trait were integrated into a consensus QTL by meta-analysis using BioMercator 2.1 software with default parameters. [46, 47], and were then named with initial letters ‘*cq*’, for instance, *cqSL-A5-2*. Consensus QTLs occurring in at least one microenvironment with PVE > 20 %, or in at least two microenvironments with PVE > 10 %, were considered as major QTLs [4]. The consensus QTLs with overlapping CIs for different traits were further integrated into one unique QTL and designated with initial letters ‘*uq*’ (for example, *uq-A5-1*). If a consensus QTL had no overlapping CI with others, it was also regarded as a unique QTL.

### Construction of the consensus map and QTL comparison

Consensus map construction and QTL comparison were carried out with BioMercator 2.1 [46, 47]. The populations chosen for comparison included three DH and two RIL populations: SS [1], BE [5], QN [6], TN [4] and HY [3] (Additional file 6). A ‘two-round’ strategy was used for QTL comparison [17]. In the first round, QTLs identified in each population were collected, and those QTLs in one population of the same trait with overlapping CIs were integrated into consensus QTLs using meta-analysis. The consensus QTLs were named with the population abbreviation followed with *‘cq*’ and the name of traits, a hyphen (-) and the linkage group. A number was added if more than one QTL was located on same chromosome (e.g. *QN-cqSL-A10-1*). In the second round, based on the common markers, the markers in homologous chromosomes were projected from other maps onto the reference KN DH map to construct a consensus map. Then the consensus QTLs detected in different populations were aligned to the KN map and QTLs were compared between published QTLs and our results.

### Map alignment of *B. napus* with *Arabidopsis*, *B. rapa* and *B. oleracea*

Among the 403 markers mapped in the KN genetic map, 141 markers with known sequence information were treated as anchor markers for map alignment between *B. napus* and *Arabidopsis* [27]. Finally, 33 blocks and 127 islands were aligned between *Arabidopsis* pseudochromosomes and the consensus KN DH linkage map [27]. The related genes that may control the seven silique traits of *Arabidopsis* on each block were located according to their physical position in the *Arabidopsis* genome*.* Their genetic position on the KN DH map was obtained according to their closest anchor markers in the same synteny block. If the aligned gene(s) was located in the CI of a QTL, then the orthologous candidate genes were assumed to be associated with the target QTL. When compared with *B. rapa* and *B. oleracea*, the related genes consisting of each chromosome were obtained based on sequence information of molecular markers. To identify and locate the putative genes in *B. napus*, the homologous sequences of genes related to silique traits in the databases of *B. rapa* and *B. oleracea* were searched with the BLASTn program (E value < 1E–20 when using *Arabidopsis* genes related to silique traits as queries). The resulting sequences from the search were first mapped onto the chromosomes of *B. rapa* or *B. oleracea* and then placed on the *B. napus* linkage groups, based on the homologous collinear relationships between *B. napus* and *B. rapa/B. oleracea via* the *Arabidopsis* genome.

### Availability of data and material

The datasets supporting the conclusions of this article are included within the article and its additional files.

## Additional files

Additional file 1: Mean value and phenotypic variation of the seven silique traits of KN population in 3/4 microenvironments. (XLSX 14 kb) Additional file 2: Linear model comparison for different traits in three microenvironments. (DOCX 2412 kb) Additional file 3: Detailed information of identified and consensus QTLs associated with the seven silique traits in the KN population. (XLSX 29 kb) Additional file 4: Number of QTLs for the seven traits and their location distribution*. (TIF 892 kb)*
Additional file 5: Detailed information of unique QTLs associated with the seven silique traits in KN population. (XLSX 37 kb) Additional file 6: Detailed information of the consensus map and QTLs collected from the five published populations. (XLSX 178 kb) Additional file 7: The consensus map and QTLs for silique related traits detected in different populations.. (PDF 7711 kb) Additional file 8: Thirty-four QTLs obtained from the five chosen maps were aligned onto the consensus map. (XLSX 12 kb) Additional file 9: Comparative mapping of homologous linkage groups between *B. napus* and *B. rapa/B. oleracea*. (DOCX 1326 kb) Additional file 10: Comparative mapping results between *Arabidopsis* and *B. napus. (XLSX 19 kb)*
Additional file 11: Function of the 25 candidate genes. (XLSX 13 kb)
