# Supplementary figures and images for: Quantitative trait loci analysis and genome-wide comparison for silique related traits in Brassica napus
Source: BMC Plant Biol. 2016 Mar 22;16:71. doi: 10.1186/s12870-016-0759-7 (PMC4802616; doi:10.1186/s12870-016-0759-7)

**Additional file 2: Linear model comparison for different traits in three microenvironments.**


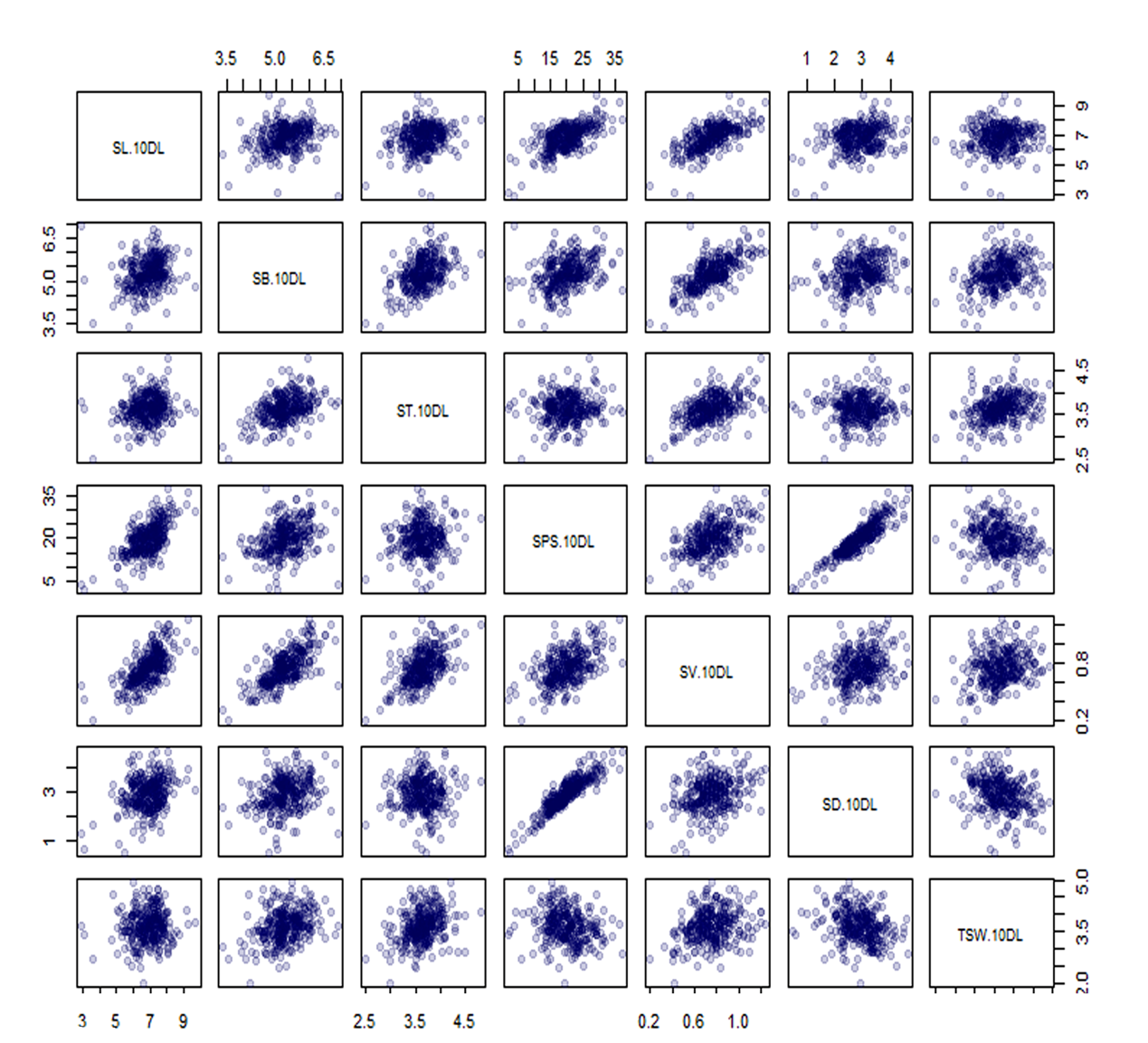

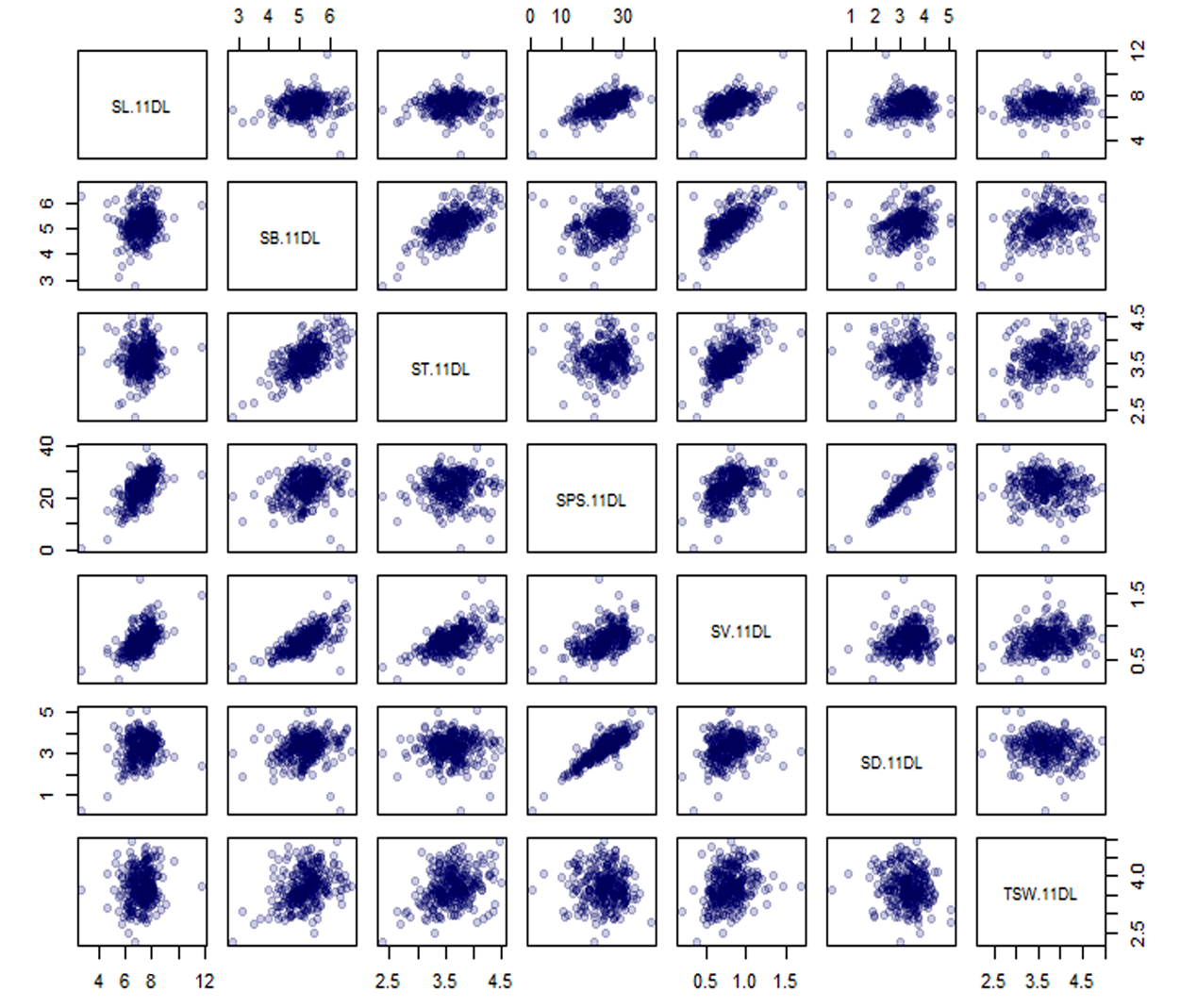

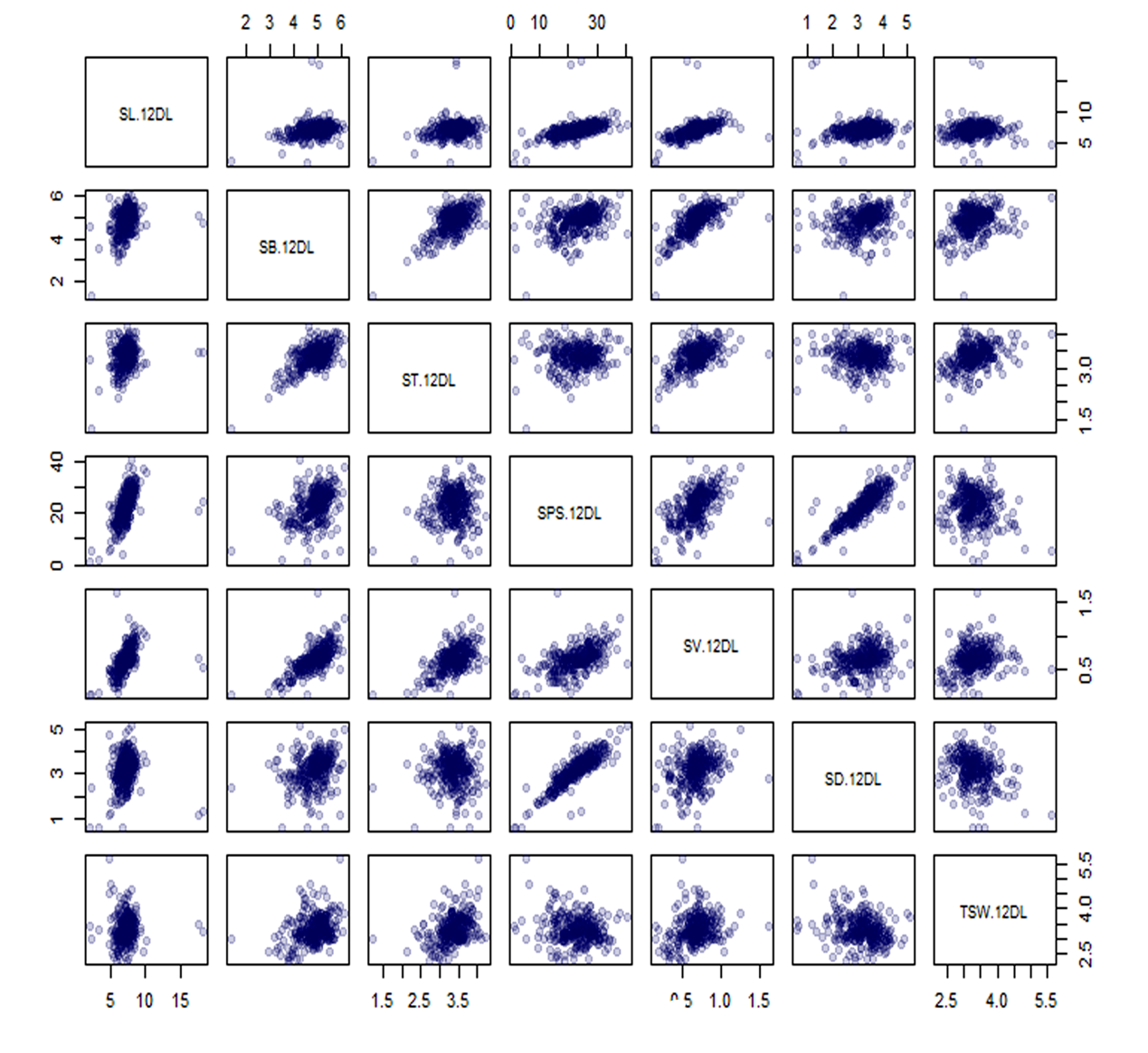

Supplement: Additional file 2: — Linear model comparison for different traits in three microenvironments. (DOCX 2412 kb) [file 12870_2016_759_MOESM2_ESM.docx]

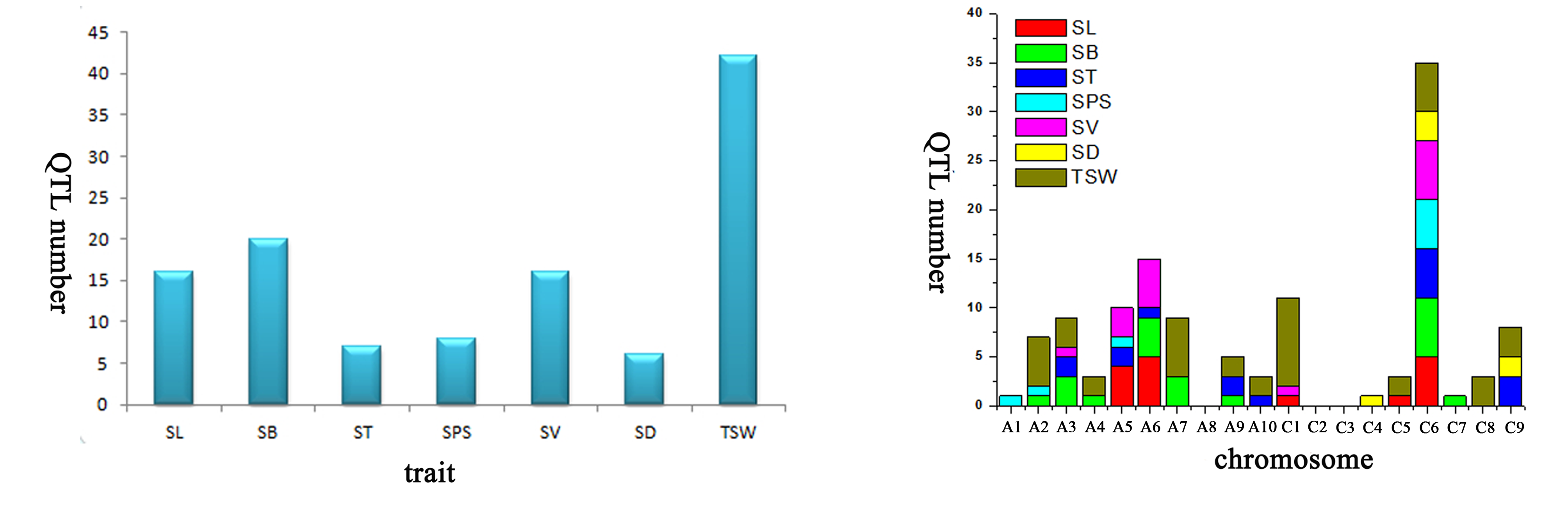

Supplement: Additional file 4: — Number of QTLs for the seven traits and their location distribution. (TIF 892 kb) [file 12870_2016_759_MOESM4_ESM.tif]
