# Supplementary material for: Quantitative trait loci analysis and genome-wide comparison for silique related traits in Brassica napus
Source: BMC Plant Biol. 2016 Mar 22;16:71. doi: 10.1186/s12870-016-0759-7 (PMC4802616; doi:10.1186/s12870-016-0759-7)
Supplement: Additional file 7: — The consensus map and QTLs for silique related traits detected in different populations.. (PDF 7711 kb) [file 12870_2016_759_MOESM7_ESM.pdf]

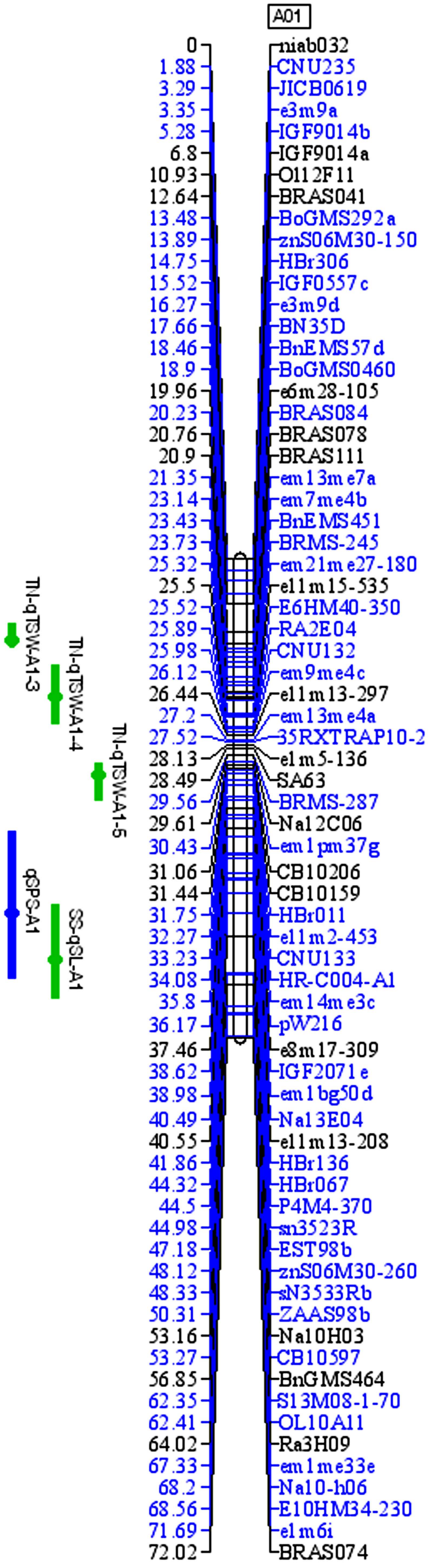
A01
0 niab032
1.88 CNU235
3.29 JICB0619
3.35 e3m9a
5.28 IGF9014b
6.8 IGF9014a
10.93 Ol12F11
12.64 BRAS041
13.48 BoGMS292a
13.89 znS06M30-150
14.75 HBr306
15.52 IGF0557c
16.27 e3m9d
17.66 BN35D
18.46 BnEMS57d
18.9 BoGMS0460
19.96 e6m28-105
20.23 BRAS084
20.76 BRAS078
20.9 BRAS111
21.35 em13me7a
23.14 em7me4b
23.43 BnEMS451
23.73 BRMS-245
25.32 em21me27-180
25.5 e11m15-535
25.52 E6HM40-350
25.89 RA2E04
25.98 CNU132
26.12 em9me4c
26.44 e11m13-297
27.2 em13me4a
27.52 35RXTRAP10-2
28.13 e1m5-136
28.49 SA63
29.56 BRMS-287
29.61 Na12C06
30.43 em1pm37g
31.06 CB10206
31.44 CB10159
31.75 HBr011
32.27 e11m2-453
33.23 CNU133
34.08 HR-C004-A1
35.8 em14me3c
36.17 pW216
37.46 e8m17-309
38.62 IGF2071e
38.98 em1bg50d
40.49 Na13E04
40.55 e11m13-208
41.86 HBr136
44.32 HBr067
44.5 P4M4-370
44.98 sn3523R
47.18 EST98b
48.12 znS06M30-260
48.33 sN3533Rb
50.31 ZAAS98b
53.16 Na10H03
53.27 CB10597
56.85 BnGMS464
62.35 S13M08-1-70
62.41 OL10A11
64.02 Ra3H09
67.33 em1me33e
68.2 Na10-h06
68.56 E10HM34-230
71.69 e1m6i
72.02 BRAS074
TN-qTSW-A1-3
TN-qTSW-A1-4
TN-qTSW-A1-5
qSPS-A1
SS-qSL-A1

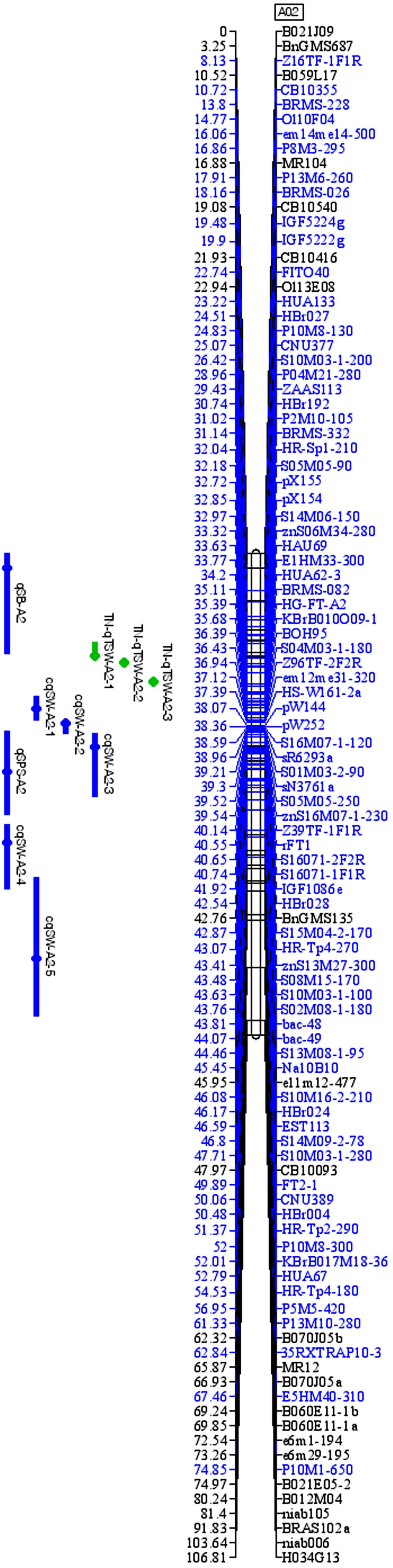
A02
0 B021J09
3.25 BnGMS687
8.13 Z16TF-1F1R
10.52 B059L17
10.72 CB10355
13.8 BRMS-228
14.77 O110F04
16.06 em14me14-500
16.86 P8M3-295
16.88 MR104
17.91 P13M6-260
18.16 BRMS-026
19.08 CB10540
19.48 IGF5224g
19.9 IGF5222g
21.93 CB10416
22.74 FITO40
22.94 O113E08
23.22 HUA133
24.51 HBr027
24.83 P10M8-130
25.07 CNU377
26.42 S10M03-1-200
28.96 P04M21-280
29.43 ZAAS113
30.74 HBr192
31.02 P2M10-105
31.14 BRMS-332
32.04 HR-Sp1-210
32.18 S05M05-90
32.72 pX155
32.85 pX154
32.97 S14M06-150
33.32 znS06M34-280
33.63 HAU69
33.77 E1HM33-300
34.2 HUA62-3
35.11 BRMS-082
35.39 HG-FT-A2
35.68 KBrB010O09-1
36.39 BOH95
36.43 S04M03-1-180
36.94 Z96TF-2F2R
37.12 em12me31-320
37.39 HS-W161-2a
38.07 pW144
38.36 pW252
38.59 S16M07-1-120
38.96 sR6293a
39.21 S01M03-2-90
39.3 sN3761a
39.52 S05M05-250
39.54 znS16M07-1-230
40.14 Z39TF-1F1R
40.55 rFT1
40.65 S16071-2F2R
40.74 S16071-1F1R
41.92 IGF1086e
42.54 HBr028
42.76 BnGMS135
42.87 S15M04-2-170
43.07 HR-Tp4-270
43.41 znS13M27-300
43.48 S08M15-170
43.63 S10M03-1-100
43.76 S02M08-1-180
43.81 bac-48
44.07 bac-49
44.46 S13M08-1-95
45.45 Na10B10
45.95 e11m12-477
46.08 S10M16-2-210
46.17 HBr024
46.59 EST113
46.8 S14M09-2-78
47.71 S10M03-1-280
47.97 CB10093
49.89 FT2-1
50.06 CNU389
50.48 HBr004
51.37 HR-Tp2-290
52 P10M8-300
52.01 KBrB017M18-36
52.79 HUA67
54.53 HR-Tp4-180
56.95 P5M5-420
61.33 P13M10-280
62.32 B070J05b
62.84 35RXTRAP10-3
65.87 MR12
66.93 B070J05a
67.46 E5HM40-310
69.24 B060E11-1b
69.85 B060E11-1a
72.54 e6m1-194
73.26 e6m29-195
74.85 P10M1-650
74.97 B021E05-2
80.24 B012M04
81.4 niab105
91.83 BRAS102a
103.64 niab006
106.81 H034G13
qSB-A2
TN-qTSW-A2-1
TN-qTSW-A2-2
TN-qTSW-A2-3
cqSW-A2-1
cqSW-A2-2
cqSW-A2-3
qSPS-A2
cqSW-A2-4
cqSW-A2-5

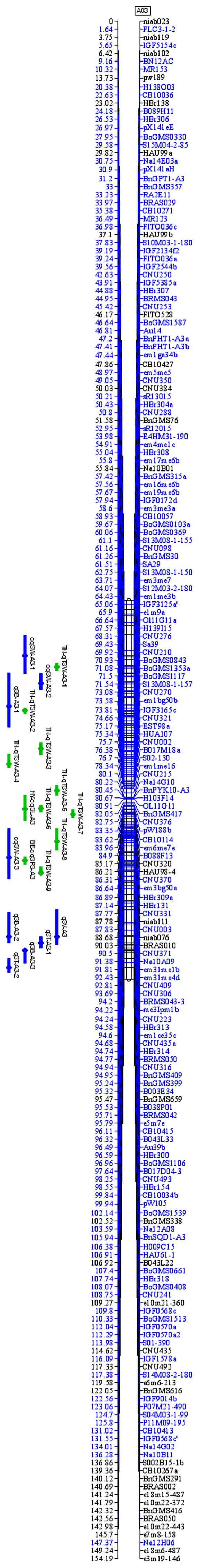

A03
0 niab023
1.64 FLC3-1-2
3.75 niab119
5.65 IGF5154c
6.42 niab102
9.16 BN12AC
10.32 MR153
13.73 pw189
20.38 H138O03
22.63 CB10036
23.02 HBr138
24.18 B089H11
26.53 HBr306
26.97 pX141eE
27.95 BoGMS0330
29.58 S15M04-2-85
29.83 HAU99e
30.75 Na14E03a
30.9 pX141aH
31.2 BnGPT1-A3
33 BnGMS357
33.23 RA2E11
33.97 BRAS029
35.38 CB10271
36.49 MR123
36.98 FITO036c
37.1 HAU99b
37.83 S10M03-1-180
39.19 IGF2134f2
39.24 FITO036a
39.56 IGF2544b
42.63 CNU250
43.91 IGF5385a
44.88 HBr307
44.95 BRMS043
45.42 CNU253
46.17 FITO528
46.64 BoGMS1587
46.81 Aul4
47.2 BnPHT1-A3a
47.41 BnPHT1-A3b
47.44 em1ga34b
47.86 CB10427
48.97 em5me5
49.05 CNU350
50.03 CNU384
50.21 sR13015
50.43 HBr304a
50.8 CNU288
51.58 BnGMS76
52.95 sR12015
53.98 E4HM31-190
54.91 em4me1c
55.04 HBr308
55.8 em17me6b
55.84 Na10B01
57.42 BnGMS315a
57.56 em16me6b
57.67 em19me6b
57.94 IGF0172d
58.6 em3me3a
58.93 CB10057
59.67 BoGMS0103a
60.06 BoGMS0369
61.1 S13M08-1-155
61.16 CNU098
61.26 BnGMS30
61.51 SA29
62.75 S13M08-1-150
63.71 em3me7
64.07 S12M03-2-180
64.43 em1me3b
65.06 IGF3125a'
65.9 e1m9a
66.64 Ol11G11a
67.57 H139I15
68.31 CNU276
69.43 Sa39
69.92 CNU210
70.93 BoGMS0843
71.08 BoGMS1353a
71.5 BoGMS1117
71.54 S13M08-1-157
73.08 CNU270
73.58 em1bg50b
73.81 IGF3165c
74.66 CNU321
75.17 EST98a
75.34 HUA107
75.7 CNU002
76.38 B017M18a
76.7 S02-130
78.34 em1me16
80.1 CNU215
80.22 Na14G10
80.45 BnPYK10-A3
80.67 H103F14
80.91 OL11G11
82.05 BnGMS417
82.6 CNU376
83.35 pW188b
83.62 CB10114
83.96 em6me7e
84.9 B088F13
85.17 CNU320
86.21 HAU98-4
86.31 CNU370
86.64 em3bg50a
86.89 HBr309a
87.14 HBr131
87.77 CNU331
87.78 niab111
87.83 CNU003
88.68 niab076
90.03 BRAS010
90.3 CNU371
91.38 Na10A09
91.81 em31me1b
92.43 em31me4d
92.81 CNU409
93.69 CNU306
94.2 BRMS043-3
94.22 me3Ipm1b
94.24 CNU223
94.58 HBr313
94.6 em1ce35c
94.68 CNU435a
94.74 HBr314
94.77 BRMS050
94.94 CNU316
94.95 BnGMS409
95.24 BnGMS399
95.32 B003E34
95.47 BnGMS659
95.53 B038P01
95.71 BRMS042
95.79 e5m7e
96.11 CB10415
96.32 B043L33
96.49 Au39b
96.59 HBr300
96.96 BoGMS1106
97.64 B017D04-3
98.25 CNU493
98.55 HBr154
99.84 CB10034b
99.94 pW105
102.14 BoGMS1539
102.52 BnGMS338
103.59 Na12A08
105.94 BnSQD1-A3
106.38 H009C15
106.91 HAU61-1
106.92 B043L22
107.4 BoGMS0661
107.74 HBr318
108.07 BoGMS0408
108.75 CNU241
109.27 e10m21-360
109.8 IGF0568c
110.33 BoGMS1513
112.04 IGF0570a
112.29 IGF0570a2
113.98 S01-390
114.62 CNU435
116.09 IGF1578a
117.33 CNU492
117.38 S14M08-2-180
119.58 e6m6-213
122.05 BnGMS616
122.56 IGF9014b
123.06 P07M21-490
124.7 S04M03-1-99
125.8 P11M09-195
131.02 CB10413
131.55 IGF0568c'
134.01 Na14G02
136.28 Na10B11
136.86 S002B15-1b
139.36 CB10267a
140.12 BnGMS291
140.69 BRAS002
141.24 e18m15-487
141.79 e10m22-372
142.32 BnGMS416
142.56 BRAS050
142.98 e10m22-443
145.7 e7m8-158
147.37 Na12H06
149.24 e16m6-487
154.19 e3m19-146
cqSW-A3-1
TN-qTSWA3-1
cqSW-A3-2
qSB-A3-1
TN-qTSWA3-2
TN-qTSWA3-3
TN-qTSWA3-4
TN-qTSWA3-5
TN-qTSWA3-6
TN-qTSWA3-7
TN-qTSWA3-8
HN-cqSL-A3
cqSW-A3-3
BE-cqSP-A3
TN-qTSWA3-9
qSB-A3-2
qSV-A3
qST-A3-1
qSB-A3-3
qST-A3-2

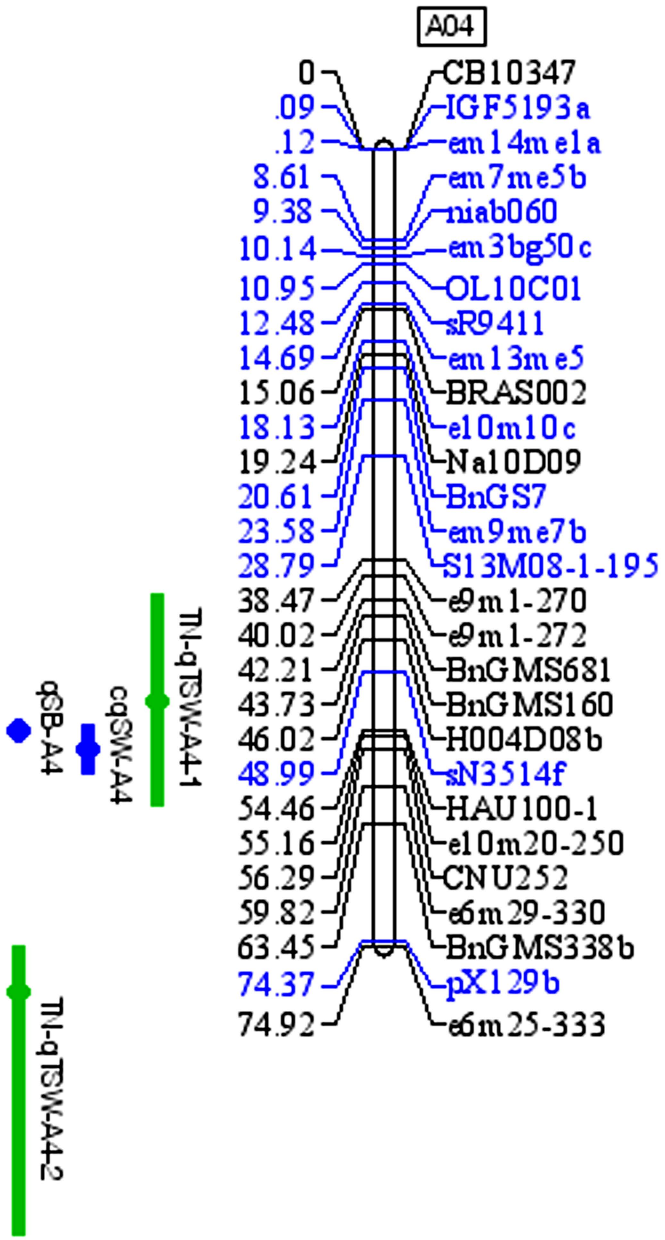
A04
0 CB10347
.09 IGF5193a
.12 em14me1a
8.61 em7me5b
9.38 niab060
10.14 em3bg50c
10.95 OL10C01
12.48 sR9411
14.69 em13me5
15.06 BRAS002
18.13 e10m10c
19.24 Na10D09
20.61 BnGS7
23.58 em9me7b
28.79 S13M08-1-195
38.47 e9m1-270
40.02 e9m1-272
42.21 BnGMS681
43.73 BnGMS160
46.02 H004D08b
48.99 sN3514f
54.46 HAU100-1
55.16 e10m20-250
56.29 CNU252
59.82 e6m29-330
63.45 BnGMS338b
74.37 pX129b
74.92 e6m25-333
TN-qTSW-A4-1
cqSW-A4
qSB-A4
TN-qTSW-A4-2

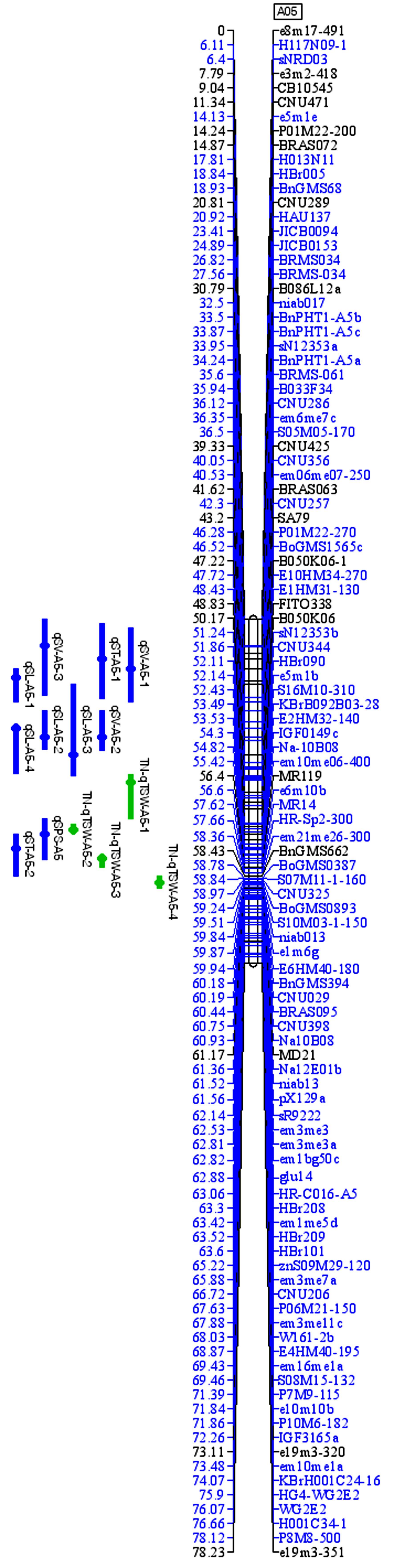
A05
0 e8m17-491
6.11 H117N09-1
6.4 sNRD03
7.79 e3m2-418
9.04 CB10545
11.34 CNU471
14.13 e5m1e
14.24 P01M22-200
14.87 BRAS072
17.81 H013N11
18.84 HBr005
18.93 BnGMS68
20.81 CNU289
20.92 HAU137
23.41 JICB0094
24.89 JICB0153
26.82 BRMS034
27.56 BRMS-034
30.79 B086L12a
32.5 niab017
33.5 BnPHT1-A5b
33.87 BnPHT1-A5c
33.95 sN12353a
34.24 BnPHT1-A5a
35.6 BRMS-061
35.94 B033F34
36.12 CNU286
36.35 em6me7c
36.5 S05M05-170
39.33 CNU425
40.05 CNU356
40.53 em06me07-250
41.62 BRAS063
42.3 CNU257
43.2 SA79
46.28 P01M22-270
46.52 BoGMS1565c
47.22 B050K06-1
47.72 E10HM34-270
48.43 E1HM31-130
48.83 FITO338
50.17 B050K06
51.24 sN12353b
51.86 CNU344
52.11 HBr090
52.14 e5m1b
52.43 S16M10-310
53.49 KBrB092B03-28
53.53 E2HM32-140
54.3 IGF0149c
54.82 Na-10B08
55.42 em10me06-400
56.4 MR119
56.6 e6m10b
57.62 MR14
57.66 HR-Sp2-300
58.36 em21me26-300
58.43 BnGMS662
58.78 BoGMS0387
58.84 S07M11-1-160
58.97 CNU325
59.24 BoGMS0893
59.51 S10M03-1-150
59.84 niab013
59.87 e1m6g
59.94 E6HM40-180
60.18 BnGMS394
60.19 CNU029
60.44 BRAS095
60.75 CNU398
60.93 Na10B08
61.17 MD21
61.36 Na12E01b
61.52 niab13
61.56 pX129a
62.14 sR9222
62.53 em3me3
62.81 em3me3a
62.82 em1bg50c
62.88 glu14
63.06 HR-C016-A5
63.3 HBr208
63.42 em1me5d
63.52 HBr209
63.6 HBr101
65.22 znS09M29-120
65.88 em3me7a
66.72 CNU206
67.63 P06M21-150
67.88 em3me11c
68.03 W161-2b
68.87 E4HM40-195
69.43 em16me1a
69.46 S08M15-132
71.39 P7M9-115
71.84 e10m10b
71.86 P10M6-182
72.26 IGF3165a
73.11 e19m3-320
73.48 em10me1a
74.07 KBrH001C24-16
75.9 HG4-WG2E2
76.07 WG2E2
76.66 H001C34-1
78.12 P8M8-500
78.23 e19m3-351
qSV-A5-3
qST-A5-1
qSV-A5-1
qSL-A5-1
qSL-A5-3
qSL-A5-2
qSV-A5-2
qSL-A5-4
TN-qTSW-A5-1
TN-qTSW-A5-2
TN-qTSW-A5-3
TN-qTSW-A5-4
qSPS-A5
qST-A5-2

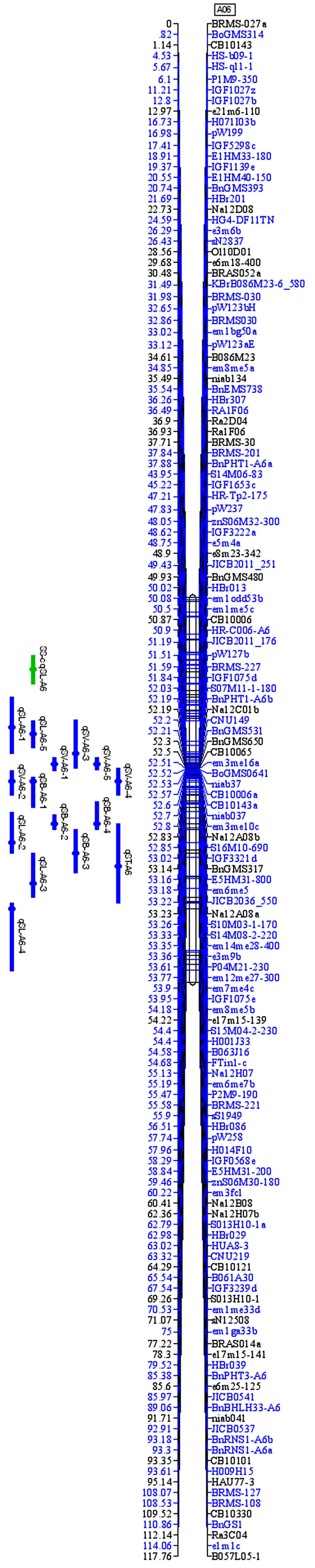
A06
0 BRMS-027a
.82 BoGMS314
1.14 CB10143
4.53 HS-b09-1
5.67 HS-q11-1
6.1 P1M9-350
11.21 IGF1027z
12.8 IGF1027b
12.97 e21m6-110
16.73 H071I03b
16.98 pW199
17.41 IGF5298c
18.91 E1HM33-180
19.37 IGF1139e
20.55 E1HM40-150
20.74 BnGMS393
21.69 HBr201
22.73 Na12D08
24.59 HG4-DF11TN
26.29 e3m6b
26.43 sN2837
28.56 Ol10D01
29.68 e6m18-400
30.48 BRAS052a
31.49 KBrB086M23-6_580
31.98 BRMS-030
32.65 pW123bH
32.86 BRMS030
33.02 em1bg50a
33.12 pW123aE
34.61 B086M23
34.85 em8me5a
35.49 niab134
35.54 BnEMS738
36.26 HBr307
36.49 RA1F06
36.9 Ra2D04
36.93 Ra1F06
37.71 BRMS-30
37.84 BRMS-201
37.88 BnPHT1-A6a
43.95 S14M06-83
45.22 IGF1653c
47.21 HR-Tp2-175
47.83 pW237
48.05 znS06M32-300
48.62 IGF3222a
48.75 e5m4a
48.9 e8m23-342
49.43 JICB2011_251
49.93 BnGMS480
50.02 HBr013
50.08 em1odd53b
50.5 em1me5c
50.87 CB10006
50.9 HR-C006-A6
51.19 JICB2011_176
51.51 pW127b
51.59 BRMS-227
51.84 IGF1075d
52.03 S07M11-1-180
52.19 BnPHT1-A6b
52.19 Na12C01b
52.2 CNU149
52.21 BnGMS531
52.3 BnGMS650
52.5 CB10065
52.51 em3me16a
52.52 BoGMS0641
52.53 niab37
52.57 CB10006a
52.6 CB10143a
52.7 niab037
52.8 em3me10c
52.83 Na12A08b
52.85 S16M10-690
53.02 IGF3321d
53.14 BnGMS317
53.16 E5HM31-800
53.18 em6me5
53.22 JICB2036_550
53.23 Na12A08a
53.26 S10M03-1-170
53.33 S14M08-2-220
53.35 em14me28-400
53.36 e3m9b
53.61 P04M21-230
53.77 em12me27-300
53.9 em7me4c
53.95 IGF1075e
54.18 em8me5b
54.22 e17m15-139
54.4 S15M04-2-230
54.4 H001J33
54.58 B063J16
54.68 FTin1-c
55.13 Na12H07
55.19 em6me7b
55.47 P2M9-190
55.58 BRMS-221
55.9 sS1949
56.51 HBr086
57.74 pW258
57.96 H014F10
58.29 IGF0568e
58.84 E5HM31-200
59.46 znS06M30-180
60.22 em3fc1
60.41 Na12B08
62.36 Na12H07b
62.79 S013H10-1a
62.98 HBr029
63.02 HUA8-3
63.32 CNU219
64.29 CB10121
65.54 B061A30
67.54 IGF3239d
69.26 S013H10-1
70.53 em1me33d
71.07 sN12508
75 em1ga33b
77.22 BRAS014a
78.3 e17m15-141
79.52 HBr039
85.38 BnPHT3-A6
85.6 e6m25-125
85.97 JICB0541
89.06 BnBHLH33-A6
91.71 niab041
92.91 JICB0537
93.18 BnRNS1-A6b
93.3 BnRNS1-A6a
93.35 CB10101
93.61 H009H15
95.14 HAU77-3
108.07 BRMS-127
108.53 BRMS-108
109.52 CB10330
110.86 BnGS1
112.14 Ra3C04
114.06 e1m1c
117.76 B057L05-1
SS-cqSL-A6
qSL-A6-1
qSL-A6-5
qSV-A6-3
qSV-A6-1
qSV-A6-5
qSV-A6-4
qSV-A6-2
qSB-A6-1
qSB-A6-2
qSB-A6-4
qSL-A6-2
qSB-A6-3
qST-A6
qSL-A6-3
qSL-A6-4

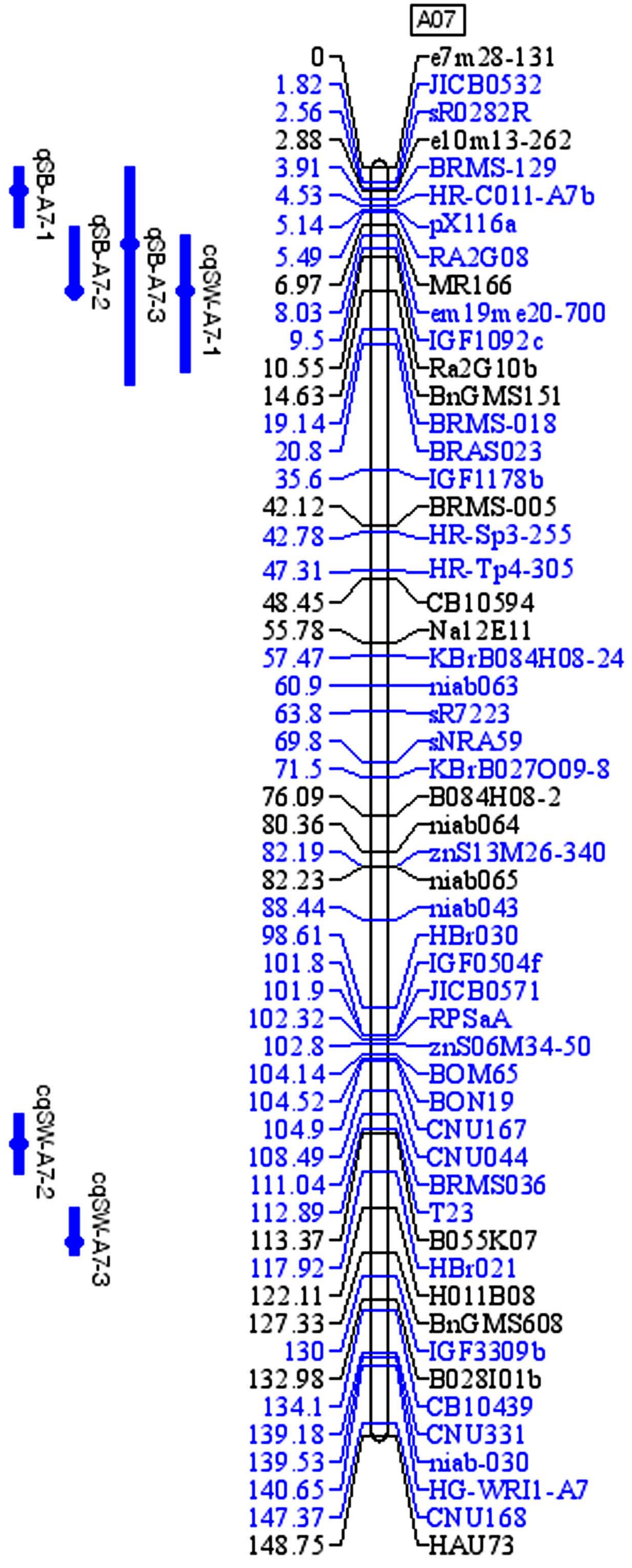
A07
0 e7m28-131
1.82 JICB0532
2.56 sR0282R
2.88 e10m13-262
3.91 BRMS-129
4.53 HR-C011-A7b
5.14 pX116a
5.49 RA2G08
6.97 MR166
8.03 em19me20-700
9.5 IGF1092c
10.55 Ra2G10b
14.63 BnGMS151
19.14 BRMS-018
20.8 BRAS023
35.6 IGF1178b
42.12 BRMS-005
42.78 HR-Sp3-255
47.31 HR-Tp4-305
48.45 CB10594
55.78 Na12E11
57.47 KBrB084H08-24
60.9 niab063
63.8 sR7223
69.8 sNRA59
71.5 KBrB027O09-8
76.09 B084H08-2
80.36 niab064
82.19 znS13M26-340
82.23 niab065
88.44 niab043
98.61 HBr030
101.8 IGF0504f
101.9 JICB0571
102.32 RPSaA
102.8 znS06M34-50
104.14 BOM65
104.52 BON19
104.9 CNU167
108.49 CNU044
111.04 BRMS036
112.89 T23
113.37 B055K07
117.92 HBr021
122.11 H011B08
127.33 BnGMS608
130 IGF3309b
132.98 B028I01b
134.1 CB10439
139.18 CNU331
139.53 niab-030
140.65 HG-WRI1-A7
147.37 CNU168
148.75 HAU73
qSB-A7-1
qSB-A7-2
qSB-A7-3
cqSW-A7-1
cqSW-A7-2
cqSW-A7-3

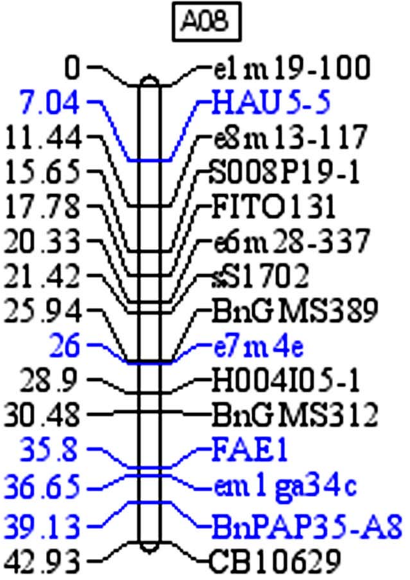
A08
0 e1m19-100
7.04 HAU5-5
11.44 e8m13-117
15.65 S008P19-1
17.78 FITO131
20.33 e6m28-337
21.42 sS1702
25.94 BnGMS389
26 e7m4e
28.9 H004I05-1
30.48 BnGMS312
35.8 FAE1
36.65 em1ga34c
39.13 BnPAP35-A8
42.93 CB10629

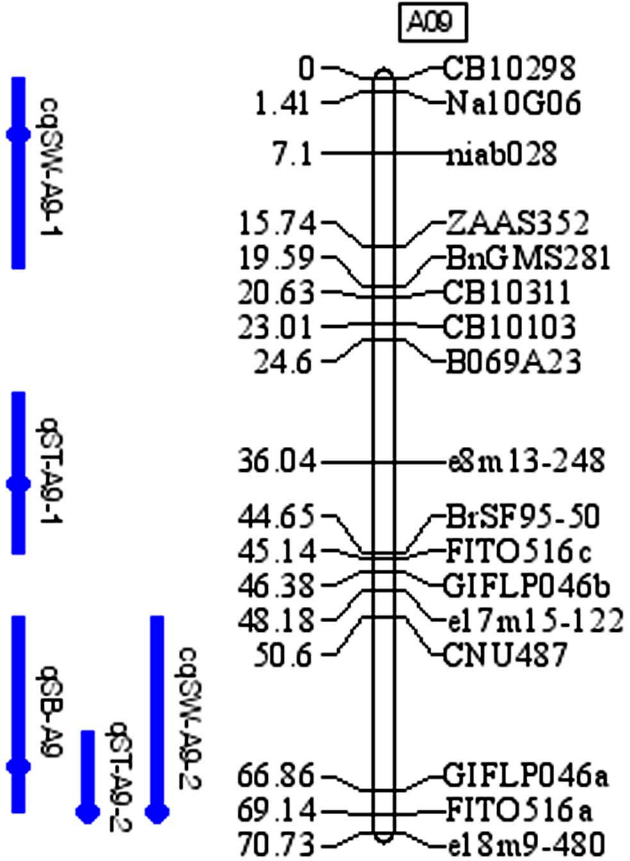

A09
0 CB10298
1.41 Na10G06
7.1 niab028
15.74 ZAAS352
19.59 BnGMS281
20.63 CB10311
23.01 CB10103
24.6 B069A23
36.04 e8m13-248
44.65 BrSF95-50
45.14 FITO516c
46.38 GIFLP046b
48.18 e17m15-122
50.6 CNU487
66.86 GIFLP046a
69.14 FITO516a
70.73 e18m9-480
cqSW-A9-1
qST-A9-1
qSB-A9
qST-A9-2
cqSW-A9-2

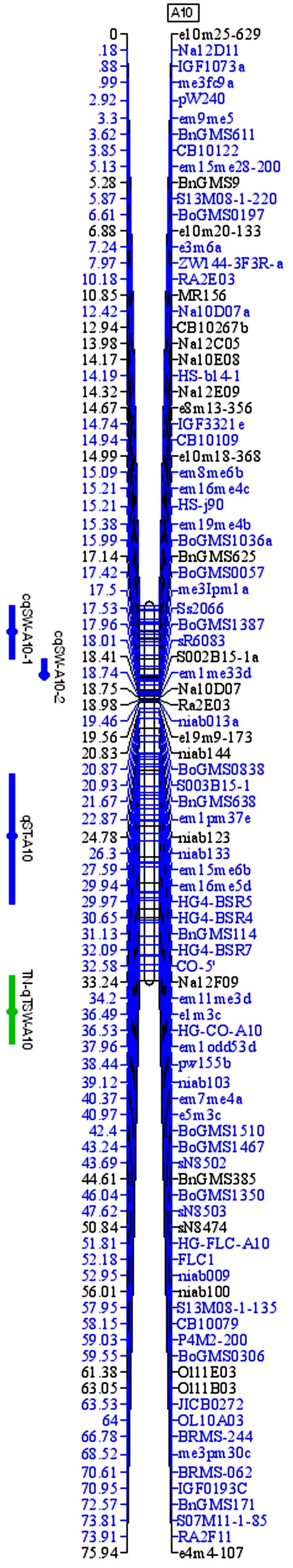
A10
0 e10m25-629
.18 Na12D11
.88 IGF1073a
.99 me3fc9a
2.92 pW240
3.3 em9me5
3.62 BnGMS611
3.85 CB10122
5.13 em15me28-200
5.28 BnGMS9
5.87 S13M08-1-220
6.61 BoGMS0197
6.88 e10m20-133
7.24 e3m6a
7.97 ZW144-3F3R-a
10.18 RA2E03
10.85 MR156
12.42 Na10D07a
12.94 CB10267b
13.98 Na12C05
14.17 Na10E08
14.19 HS-b14-1
14.32 Na12E09
14.67 e8m13-356
14.74 IGF3321e
14.94 CB10109
14.99 e10m18-368
15.09 em8me6b
15.21 em16me4c
15.21 HS-j90
15.38 em19me4b
15.99 BoGMS1036a
17.14 BnGMS625
17.42 BoGMS0057
17.5 me3Ipm1a
17.53 Ss2066
17.96 BoGMS1387
18.01 sR6083
18.41 S002B15-1a
18.74 em1me33d
18.75 Na10D07
18.98 Ra2E03
19.46 niab013a
19.56 e19m9-173
20.83 niab144
20.87 BoGMS0838
20.93 S003B15-1
21.67 BnGMS638
22.87 em1pm37e
24.78 niab123
26.3 niab133
27.59 em15me6b
29.94 em16me5d
29.97 HG4-BSR5
30.65 HG4-BSR4
31.13 BnGMS114
32.09 HG4-BSR7
32.58 CO-5'
33.24 Na12F09
34.2 em11me3d
36.49 e1m3c
36.53 HG-CO-A10
37.96 em1odd53d
38.44 pw155b
39.12 niab103
40.37 em7me4a
40.97 e5m3c
42.4 BoGMS1510
43.24 BoGMS1467
43.69 sN8502
44.61 BnGMS385
46.04 BoGMS1350
47.62 sN8503
50.84 sN8474
51.81 HG-FLC-A10
52.18 FLC1
52.95 niab009
56.01 niab100
57.95 S13M08-1-135
58.15 CB10079
59.03 P4M2-200
59.55 BoGMS0306
61.38 Ol11E03
63.05 Ol11B03
63.53 JICB0272
64 OL10A03
66.78 BRMS-244
68.52 me3pm30c
70.61 BRMS-062
70.95 IGF0193C
72.57 BnGMS171
73.81 S07M11-1-85
73.91 RA2F11
75.94 e4m4-107
cqSW-A10-1
cqSW-A10-2
qST-A10
TN-qTSW-A10

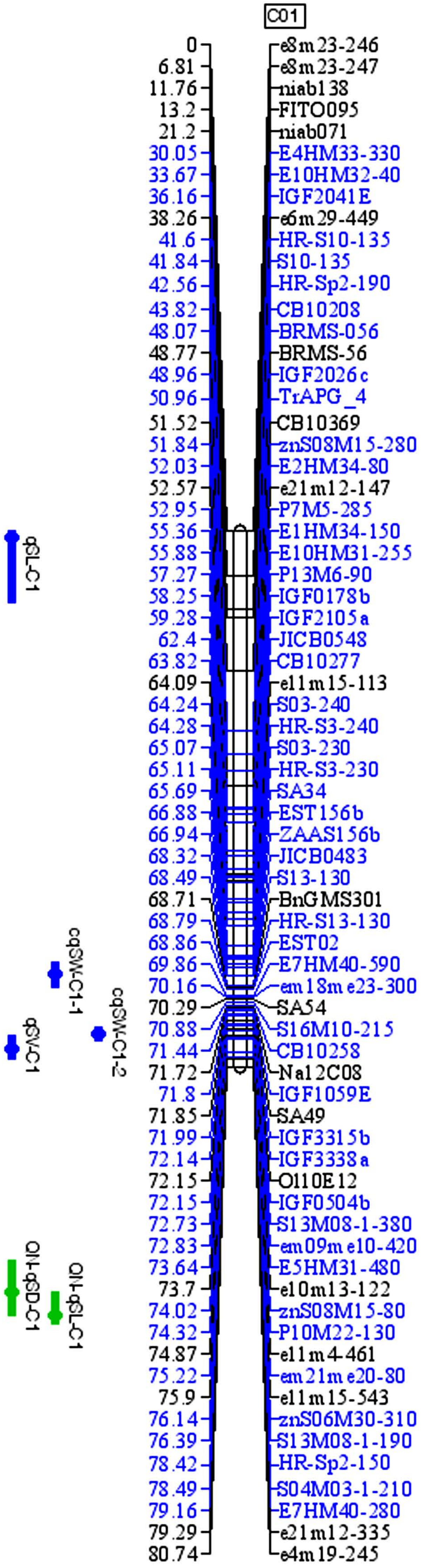

C01
0 e8m23-246
6.81 e8m23-247
11.76 niab138
13.2 FITO095
21.2 niab071
30.05 E4HM33-330
33.67 E10HM32-40
36.16 IGF2041E
38.26 e6m29-449
41.6 HR-S10-135
41.84 S10-135
42.56 HR-Sp2-190
43.82 CB10208
48.07 BRMS-056
48.77 BRMS-56
48.96 IGF2026c
50.96 TrAPG_4
51.52 CB10369
51.84 znS08M15-280
52.03 E2HM34-80
52.57 e21m12-147
52.95 P7M5-285
55.36 E1HM34-150
55.88 E10HM31-255
57.27 P13M6-90
58.25 IGF0178b
59.28 IGF2105a
62.4 JICB0548
63.82 CB10277
64.09 e11m15-113
64.24 S03-240
64.28 HR-S3-240
65.07 S03-230
65.11 HR-S3-230
65.69 SA34
66.88 EST156b
66.94 ZAAS156b
68.32 JICB0483
68.49 S13-130
68.71 BnGMS301
68.79 HR-S13-130
68.86 EST02
69.86 E7HM40-590
70.16 em18me23-300
70.29 SA54
70.88 S16M10-215
71.44 CB10258
71.72 Na12C08
71.8 IGF1059E
71.85 SA49
71.99 IGF3315b
72.14 IGF3338a
72.15 Ol10E12
72.15 IGF0504b
72.73 S13M08-1-380
72.83 em09me10-420
73.64 E5HM31-480
73.7 e10m13-122
74.02 znS08M15-80
74.32 P10M22-130
74.87 e11m4-461
75.22 em21me20-80
75.9 e11m15-543
76.14 znS06M30-310
76.39 S13M08-1-190
78.42 HR-Sp2-150
78.49 S04M03-1-210
79.16 E7HM40-280
79.29 e21m12-335
80.74 e4m19-245
qSL-C1
cqSW-C1-1
cqSW-C1-2
qSW-C1
OIl-qSD-C1
OIl-qSL-C1

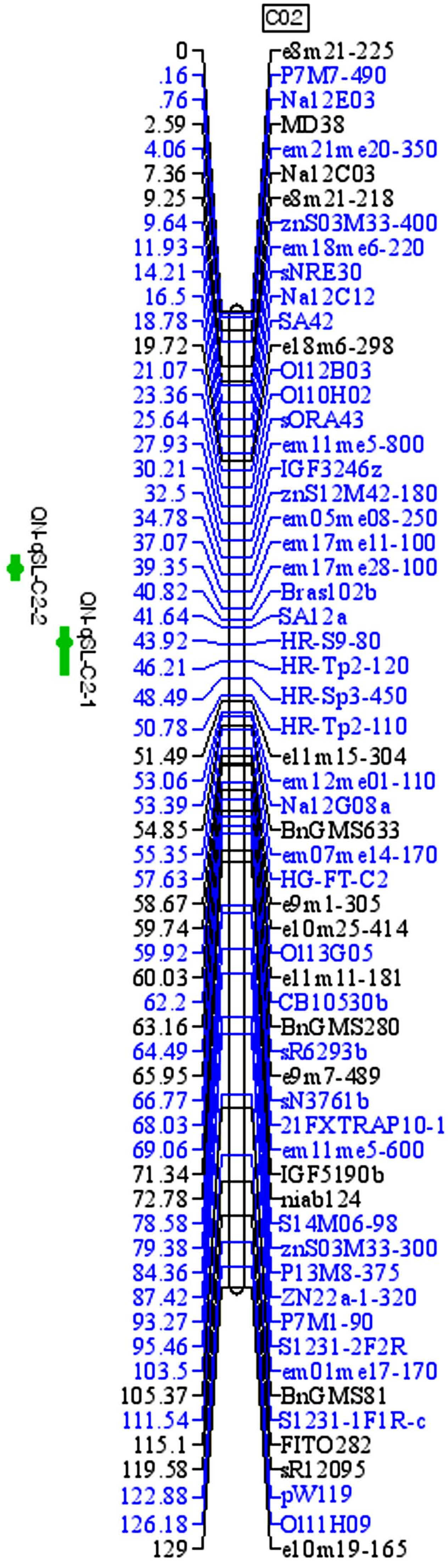
C02
QN-qSL-C2-2
QN-qSL-C2-1
0 e8m21-225
.16 P7M7-490
.76 Na12E03
2.59 MD38
4.06 em21me20-350
7.36 Na12C03
9.25 e8m21-218
9.64 znS03M33-400
11.93 em18me6-220
14.21 sNRE30
16.5 Na12C12
18.78 SA42
19.72 e18m6-298
21.07 Ol12B03
23.36 Ol10H02
25.64 sORA43
27.93 em11me5-800
30.21 IGF3246z
32.5 znS12M42-180
34.78 em05me08-250
37.07 em17me11-100
39.35 em17me28-100
40.82 Bras102b
41.64 SA12a
43.92 HR-S9-80
46.21 HR-Tp2-120
48.49 HR-Sp3-450
50.78 HR-Tp2-110
51.49 e11m15-304
53.06 em12me01-110
53.39 Na12G08a
54.85 BnGMS633
55.35 em07me14-170
57.63 HG-FT-C2
58.67 e9m1-305
59.74 e10m25-414
59.92 Ol13G05
60.03 e11m11-181
62.2 CB10530b
63.16 BnGMS280
64.49 sR6293b
65.95 e9m7-489
66.77 sN3761b
68.03 21FXTRAP10-1
69.06 em11me5-600
71.34 IGF5190b
72.78 niab124
78.58 S14M06-98
79.38 znS03M33-300
84.36 P13M8-375
87.42 ZN22a-1-320
93.27 P7M1-90
95.46 S1231-2F2R
103.5 em01me17-170
105.37 BnGMS81
111.54 S1231-1F1R-c
115.1 FITO282
119.58 sR12095
122.88 pW119
126.18 Ol11H09
129 e10m19-165

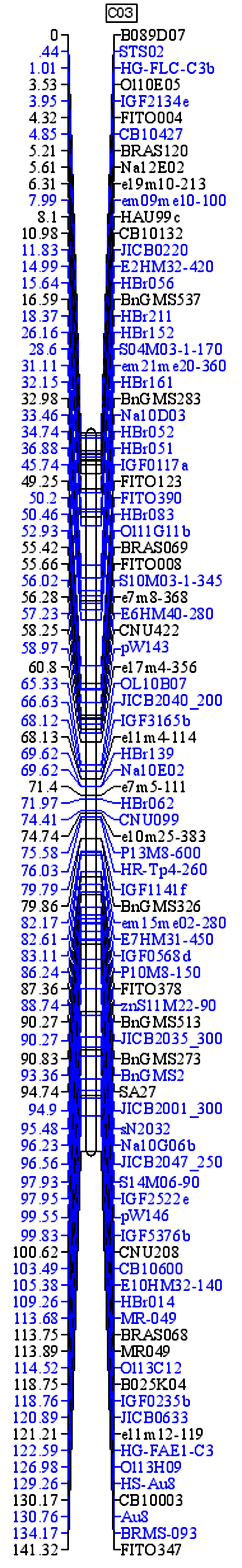

C03
0 B089D07
.44 STS02
1.01 HG-FLC-C3b
3.53 O110E05
3.95 IGF2134e
4.32 FITO004
4.85 CB10427
5.21 BRAS120
5.61 Na12E02
6.31 e19m10-213
7.99 em09me10-100
8.1 HAU99c
10.98 CB10132
11.83 JICB0220
14.99 E2HM32-420
15.64 HBr056
16.59 BnGMS537
18.37 HBr211
26.16 HBr152
28.6 S04M03-1-170
31.11 em21me20-360
32.15 HBr161
32.98 BnGMS283
33.46 Na10D03
34.74 HBr052
36.88 HBr051
45.74 IGF0117a
49.25 FITO123
50.2 FITO390
50.46 HBr083
52.93 O111G11b
55.42 BRAS069
55.66 FITO008
56.02 S10M03-1-345
56.28 e7m8-368
57.23 E6HM40-280
58.25 CNU422
58.97 pW143
60.8 e17m4-356
65.33 OL10B07
66.63 JICB2040_200
68.12 IGF3165b
68.13 e11m4-114
69.62 HBr139
69.62 Na10E02
71.4 e7m5-111
71.97 HBr062
74.41 CNU099
74.74 e10m25-383
75.58 P13M8-600
76.03 HR-Tp4-260
79.79 IGF1141f
79.86 BnGMS326
82.17 em15me02-280
82.61 E7HM31-450
83.11 IGF0568d
86.24 P10M8-150
87.36 FITO378
88.74 znS11M22-90
90.27 BnGMS513
90.27 JICB2035_300
90.83 BnGMS273
93.36 BnGMS2
94.74 SA27
94.9 JICB2001_300
95.48 sN2032
96.23 Na10G06b
96.56 JICB2047_250
97.93 S14M06-90
97.95 IGF2522e
99.55 pW146
99.83 IGF5376b
100.62 CNU208
103.49 CB10600
105.38 E10HM32-140
109.26 HBr014
113.68 MR-049
113.75 BRAS068
113.89 MR049
114.52 O113C12
118.75 B025K04
118.76 IGF0235b
120.89 JICB0633
121.21 e11m12-119
122.59 HG-FAE1-C3
126.98 O113H09
129.26 HS-Au8
130.17 CB10003
130.76 Au8
134.17 BRMS-093
141.32 FITO347

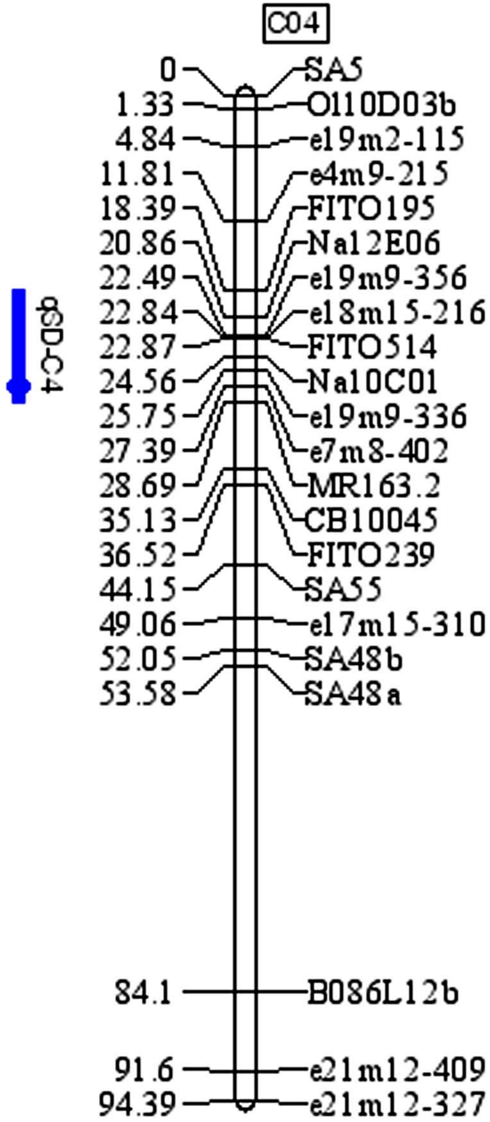
C04
0 SA5
1.33 Ol10D03b
4.84 e19m2-115
11.81 e4m9-215
18.39 FITO195
20.86 Na12E06
22.49 e19m9-356
22.84 e18m15-216
22.87 FITO514
24.56 Na10C01
25.75 e19m9-336
27.39 e7m8-402
28.69 MR163.2
35.13 CB10045
36.52 FITO239
44.15 SA55
49.06 e17m15-310
52.05 SA48b
53.58 SA48a
84.1 B086L12b
91.6 e21m12-409
94.39 e21m12-327
qSD-C4

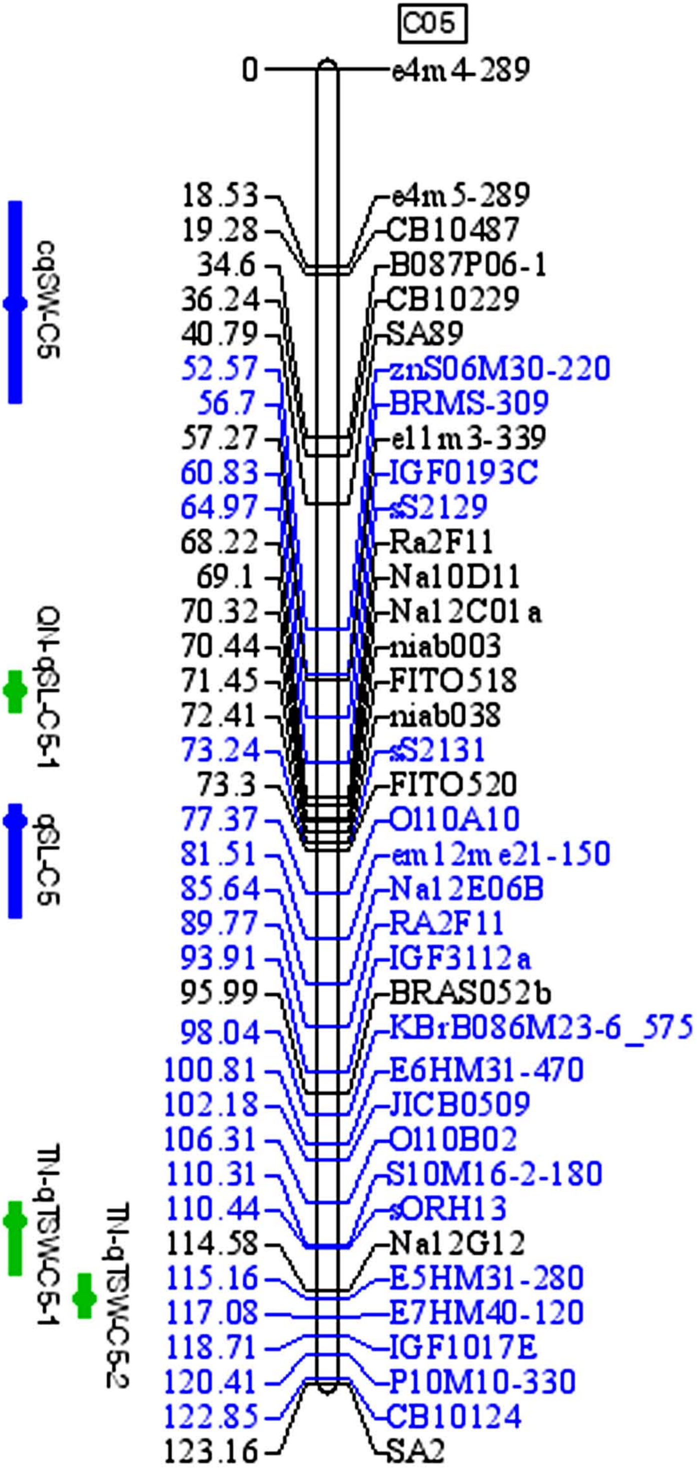

C05
0 e4m4-289
18.53 e4m5-289
19.28 CB10487
34.6 B087P06-1
36.24 CB10229
40.79 SA89
52.57 znS06M30-220
56.7 BRMS-309
57.27 e11m3-339
60.83 IGF0193C
64.97 sS2129
68.22 Ra2F11
69.1 Na10D11
70.32 Na12C01a
70.44 niab003
71.45 FITO518
72.41 niab038
73.24 sS2131
73.3 FITO520
77.37 O110A10
81.51 em12me21-150
85.64 Na12E06B
89.77 RA2F11
93.91 IGF3112a
95.99 BRAS052b
98.04 KBrB086M23-6_575
100.81 E6HM31-470
102.18 JICB0509
106.31 O110B02
110.31 S10M16-2-180
110.44 sORH13
114.58 Na12G12
115.16 E5HM31-280
117.08 E7HM40-120
118.71 IGF1017E
120.41 P10M10-330
122.85 CB10124
123.16 SA2
cqSW-C5
QN-qSL-C5-1
qSL-C5
TN-qTSW-C5-1
TN-qTSW-C5-2

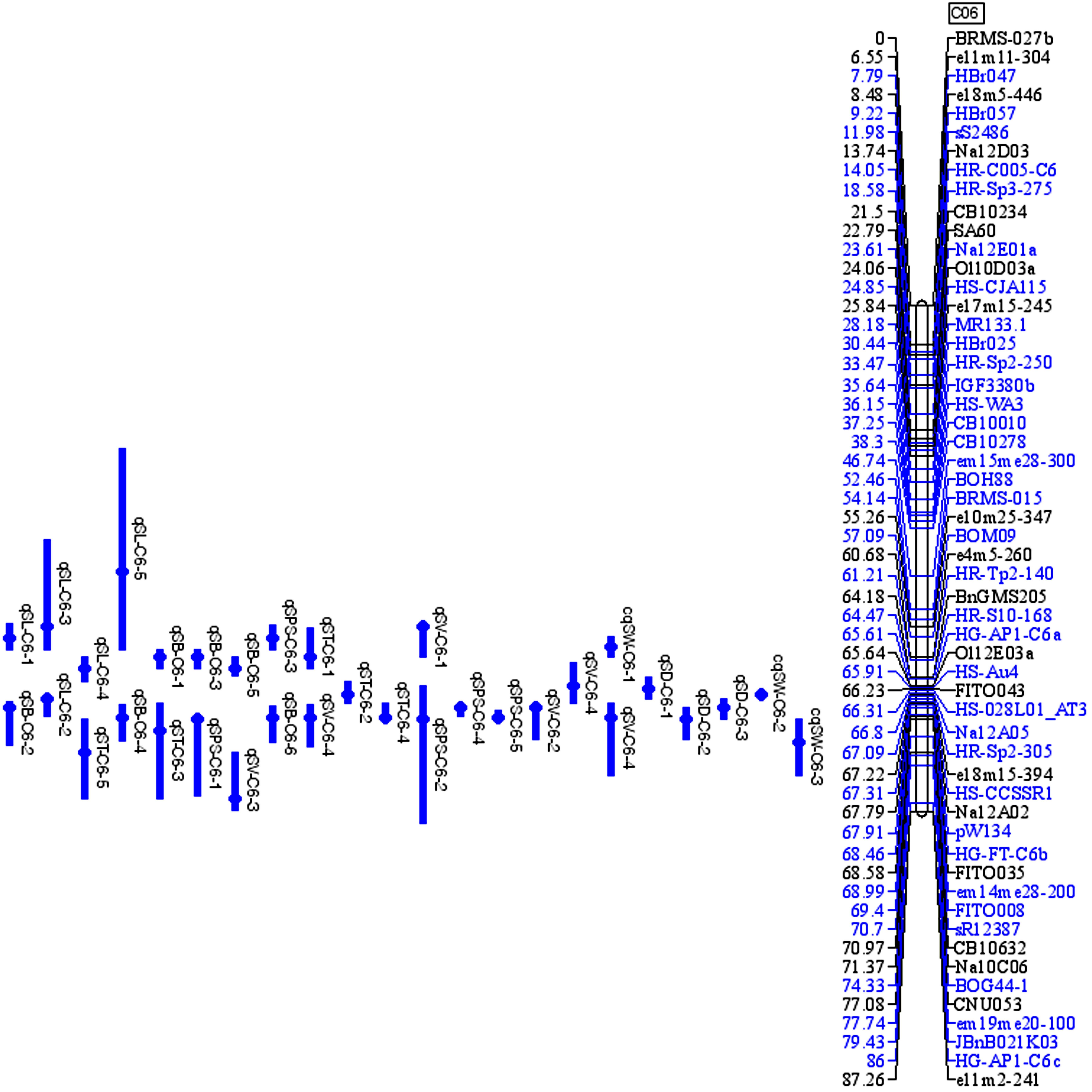
C06
0 BRMS-027b
6.55 e11m11-304
7.79 HBr047
8.48 e18m5-446
9.22 HBr057
11.98 sS2486
13.74 Na12D03
14.05 HR-C005-C6
18.58 HR-Sp3-275
21.5 CB10234
22.79 SA60
23.61 Na12E01a
24.06 Ol10D03a
24.85 HS-CJA115
25.84 e17m15-245
28.18 MR133.1
30.44 HBr025
33.47 HR-Sp2-250
35.64 IGF3380b
36.15 HS-WA3
37.25 CB10010
38.3 CB10278
46.74 em15me28-300
52.46 BOH88
54.14 BRMS-015
55.26 e10m25-347
57.09 BOM09
60.68 e4m5-260
61.21 HR-Tp2-140
64.18 BnGMS205
64.47 HR-S10-168
65.61 HG-AP1-C6a
65.64 Ol12E03a
65.91 HS-Au4
66.23 FITO043
66.31 HS-028L01_AT3
66.8 Na12A05
67.09 HR-Sp2-305
67.22 e18m15-394
67.31 HS-CCSSR1
67.79 Na12A02
67.91 pW134
68.46 HG-FT-C6b
68.58 FITO035
68.99 em14me28-200
69.4 FITO008
70.7 sR12387
70.97 CB10632
71.37 Na10C06
74.33 BOG44-1
77.08 CNU053
77.74 em19me20-100
79.43 JBnB021K03
86 HG-AP1-C6c
87.26 e11m2-241
qSL-C6-1
qSB-C6-2
qSL-C6-3
qSL-C6-2
qSL-C6-4
qST-C6-5
qSL-C6-5
qSB-C6-4
qSB-C6-1
qST-C6-3
qSB-C6-3
qSPS-C6-1
qSB-C6-5
qSV-C6-3
qSPS-C6-3
qSB-C6-6
qST-C6-1
qSV-C6-4
qST-C6-2
qST-C6-4
qSV-C6-1
qSPS-C6-2
qSPS-C6-4
qSPS-C6-5
qSV-C6-2
qSV-C6-4
cqSW-C6-1
qSV-C6-4
qSD-C6-1
qSD-C6-2
qSD-C6-3
cqSW-C6-2
cqSW-C6-3

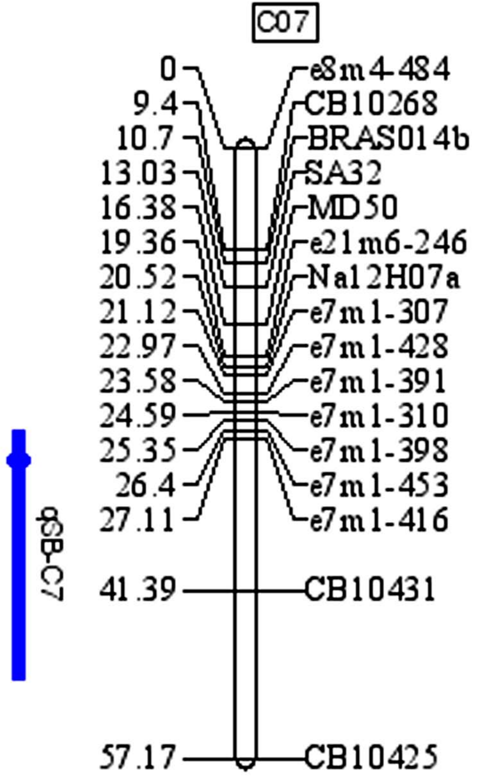

C07
0 e8m4-484
9.4 CB10268
10.7 BRAS014b
13.03 SA32
16.38 MD50
19.36 e21m6-246
20.52 Na12H07a
21.12 e7m1-307
22.97 e7m1-428
23.58 e7m1-391
24.59 e7m1-310
25.35 e7m1-398
26.4 e7m1-453
27.11 e7m1-416
41.39 CB10431
57.17 CB10425
qSB-C7

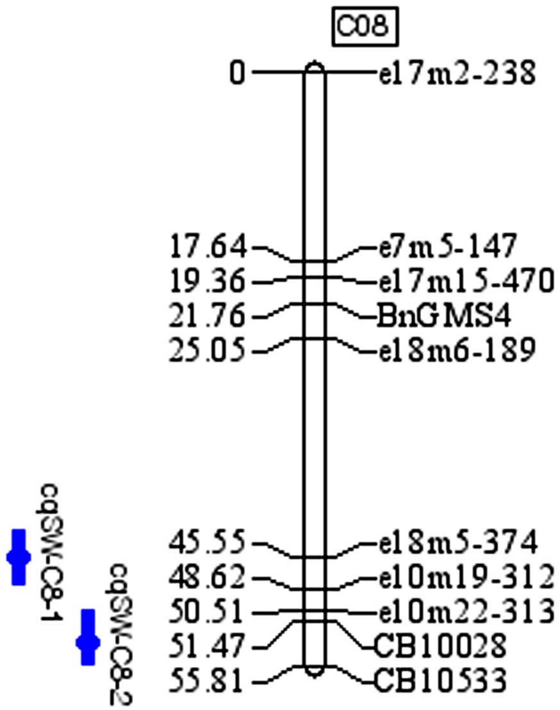
C08
0
e17m2-238
17.64
e7m5-147
19.36
e17m15-470
21.76
BnGMS4
25.05
e18m6-189
45.55
e18m5-374
48.62
e10m19-312
50.51
e10m22-313
51.47
CB10028
55.81
CB10533
cqSW-C8-1
cqSW-C8-2

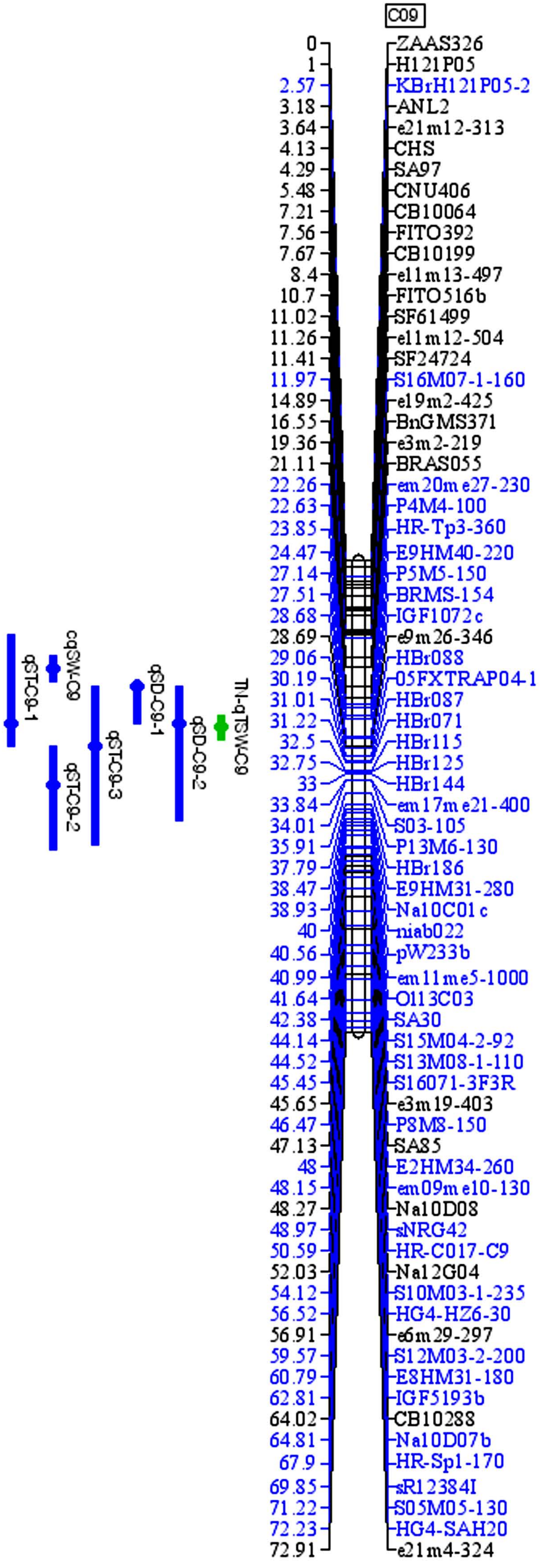
C09
0 ZAAS326
1 H121P05
2.57 KBrH121P05-2
3.18 ANL2
3.64 e21m12-313
4.13 CHS
4.29 SA97
5.48 CNU406
7.21 CB10064
7.56 FITO392
7.67 CB10199
8.4 e11m13-497
10.7 FITO516b
11.02 SF61499
11.26 e11m12-504
11.41 SF24724
11.97 S16M07-1-160
14.89 e19m2-425
16.55 BnGMS371
19.36 e3m2-219
21.11 BRAS055
22.26 em20me27-230
22.63 P4M4-100
23.85 HR-Tp3-360
24.47 E9HM40-220
27.14 P5M5-150
27.51 BRMS-154
28.68 IGF1072c
28.69 e9m26-346
29.06 HBr088
30.19 05FXTRAP04-1
31.01 HBr087
31.22 HBr071
32.5 HBr115
32.75 HBr125
33 HBr144
33.84 em17me21-400
34.01 S03-105
35.91 P13M6-130
37.79 HBr186
38.47 E9HM31-280
38.93 Na10C01c
40 niab022
40.56 pW233b
40.99 em11me5-1000
41.64 Ol13C03
42.38 SA30
44.14 S15M04-2-92
44.52 S13M08-1-110
45.45 S16071-3F3R
45.65 e3m19-403
46.47 P8M8-150
47.13 SA85
48 E2HM34-260
48.15 em09me10-130
48.27 Na10D08
48.97 sNRG42
50.59 HR-C017-C9
52.03 Na12G04
54.12 S10M03-1-235
56.52 HG4-HZ6-30
56.91 e6m29-297
59.57 S12M03-2-200
60.79 E8HM31-180
62.81 IGF5193b
64.02 CB10288
64.81 Na10D07b
67.9 HR-Sp1-170
69.85 sR12384I
71.22 S05M05-130
72.23 HG4-SAH20
72.91 e21m4-324
qST-C9-1
cqSW-C9
qST-C9-2
qST-C9-3
qSD-C9-1
qSD-C9-2
TN-qTSW-C9

Additional file 7. The consensus map and QTLs for silique related traits detected in different populations. Markers with blue color indicated these makers were projected from other maps on the KN map, *via* application of the homothetic projection based on common markers by BioMercator 2.1 software.
