# Supplementary material for: Quantitative trait loci analysis and genome-wide comparison for silique related traits in Brassica napus
Source: BMC Plant Biol. 2016 Mar 22;16:71. doi: 10.1186/s12870-016-0759-7 (PMC4802616; doi:10.1186/s12870-016-0759-7)
Supplement: Additional file 9: — Comparative mapping of homologous linkage groups between B. napus and B. rapa/B. oleracea. (DOCX 1326 kb) [file 12870_2016_759_MOESM9_ESM.docx]

**Additional file 9**: Comparative mapping of homologous linkage groups between *B. napus* and *B. rapa/B. oleracea*.


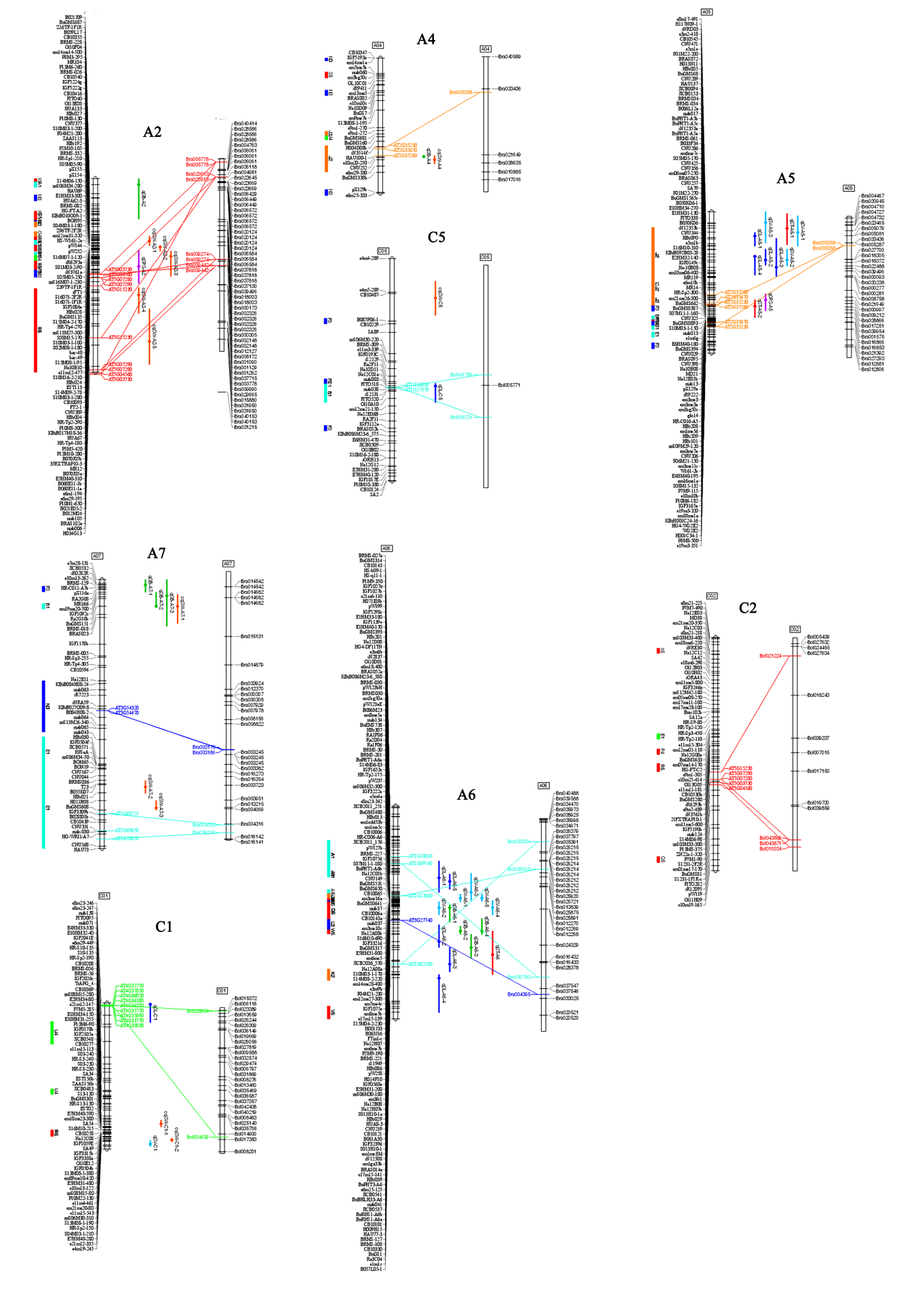


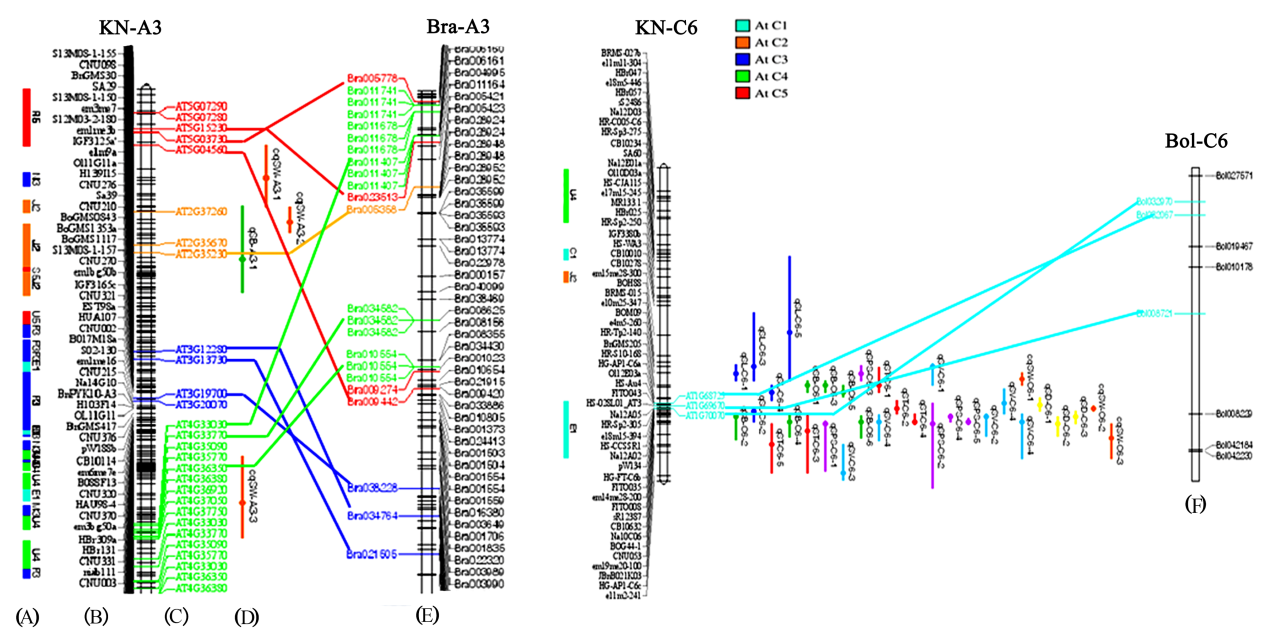


An example of comparative mapping among *B. napus*, *Arabidopsis*, *B. rapa* and *B. oleracea.* (A) the blocks or islands of *B.napus* compared with *Arabidopsis*: five colours represent the five chromosomes of *Arabidopsis*. For example, AtC1 (cyan colour) represents the first chromosome of *Arabidopsis*;(B) Adjoin the Blocks are the 19 chromosomes of *B.napus;* (C) To the right of the *B. napus* chromosomes are aligned candidate genes of *Arabidopsis*. Colours of these candidate genes are based on the block to which they belong; (D) bars with colours in the middle represent QTLs of silique related traits; (E) and (F): The section to the right of the QTLs indicates chromosomes of *B. rapa* and *B. oleracea.* Genes to the left of *B. rapa* or *B. oleracea* chromosomes are homologous genes of candidate genes. Genes to the right of *B. rapa* or *B. oleracea* chromosomes are homologous genes identified according to the sequence information of molecular markers of *B.napus* chromosomes*.* The line with colours represents one-to-one correlation between candidate genes and homologous genes of *B.rapa* or *B. oleracea.* Lines were coloured according to their related *Arabidopsis* genes.
